# Loss of Drosophila nucleostemin 1 disrupts ribosomal protein homeostasis and rRNA processing to trigger apoptosis via the Xrp1/Irbp18 complex

**DOI:** 10.1038/s41420-026-03205-9

**Published:** 2026-06-14

**Authors:** Xiuxiu Liu, Min Hou, Yuanyuan Zhang, Huan Liu, Xiaojiao Li, Yuanlin Su, Huiyuan Ding, Yanqin Gong, Qiao Liu, Yaoqin Gong, Gongping Sun

**Affiliations:** 1 Key Laboratory for Experimental Teratology of Ministry of Education, Shandong Key Laboratory of Mental Disorders and Intelligent Control, Department of Histology and Embryology, School of Basic Medical Sciences, Cheeloo College of Medicine, Shandong University, Jinan, Shandong China; 2 Key Laboratory for Experimental Teratology of Ministry of Education, and Department of Genetics, School of Basic Medical Sciences, Cheeloo College of Medicine, Shandong University, Jinan, China

**Keywords:** Apoptosis, Experimental organisms

## Abstract

Ribosome biogenesis, a process of producing ribosomes, plays an essential role in cell survival, proliferation and apoptosis. Nucleostemin (NS) is a conserved nucleolar protein highly expressed in proliferating cells but markedly reduced in differentiated cells. Previous studies on mammalian systems have shown that loss of NS triggers p53-dependent apoptosis and impairs ribosomal RNA (rRNA) processing. In this study, using the Drosophila wing imaginal disc, which is a proliferating epithelial tissue, we demonstrate that depletion of Ns1, the Drosophila homolog of mammalian NS, induces extensive p53-independent apoptosis and suppresses global translation. Notably, we find that Ns1 is required not only for rRNA processing but also for maintaining global ribosomal protein (RP) abundance. Loss of Ns1 leads to a reduction in 65 RPs. Furthermore, we show that Ns1 deficiency upregulates the Xrp1/Irbp18 complex, an effector mediating cellular response to ribosomal deficiency, which in turn activates JNK to trigger apoptosis and suppresses global translation through a mechanism independent of eIF2α phosphorylation and JNK. Our work establishes Ns1 as a critical regulator of ribosome biogenesis and cell survival in proliferating epithelia.

## Introduction

The ribosome is a macromolecular machine present in all living cells and serves as the primary site for translation. It consists of a 40S small subunit and a 60S large subunit, both composed of ribosomal RNAs (rRNAs) and numerous ribosomal proteins (RPs). Ribosome biogenesis is a highly coordinated process that largely takes place within the nucleolus. There, rRNAs are transcribed, processed and assembled with RPs to form pre-60S and pre-40S particles. These precursor particles are then exported to the cytoplasm, where the final assembly and maturation events occur. Ribosome biogenesis plays essential roles in cellular and developmental processes [1–3]. Abnormal ribosome assembly or function, arising from mutations in genes encoding RPs, rRNAs, or essential biogenesis factors, leads to ribosomopathies in humans characterized by reduced blood cells, predisposition to cancer, skeletal abnormalities, and growth retardation [4, 5].

Nucleostemin (NS) is a nucleolar GTPase highly expressed in stem cells and progenitor cells during animal development. It is required for mouse embryogenesis and for maintaining the self-renewal capacity of stem cells [6–9]. In many adult tissues, NS expression is low in differentiated cells. However, it has been reported to be upregulated following injury in several tissues and required for mouse liver regeneration [10–13]. NS is also highly expressed in many types of cancer, such as breast cancer, colon cancer, hepatocellular carcinoma, and glioma, to promote cancer cell survival, proliferation, migration, and therapy resistance [14–20]. Mechanistically, NS facilitates cell cycle progression by regulating p53, p27, ARF [20–23]. Additionally, it helps protect proliferating cells from replication-induced DNA damage by regulating the recruitment of an important repair protein, RAD51, to damage sites [13, 24, 25].

NS primarily localizes in the nucleolus, where rRNA is transcribed, processed, and assembled with RPs. Studies in *C. elegans*, zebrafish embryos, and human cells have shown that loss of NS impairs rRNA processing and/or generation of the 60S subunit [26–28]. Thus, NS is considered a regulator of ribosome biogenesis with the regulatory mechanisms unelucidated. Drosophila possesses four NS family proteins, Ns1, Ns2, Ns3, and Ns4. Among them, Ns1 is the closest ortholog to human NS. Rosby et al. have demonstrated that ubiquitous knockdown of *Ns1* in Drosophila causes larval lethality, with a few adult escapers showing defects in head, eye, and antenna. Reduced Ns1 expression impairs the proliferation of midgut imaginal island cells and the growth of polyploid cells in salivary glands. Their study also reported a role of Ns1 in ribosome biogenesis, but not in rRNA processing. They found that loss of Ns1 in polyploid cells leads to reduced ribosomes in the cytoplasm and nucleolar accumulation of overexpressed RpL11 and RpL26, suggesting a blockade in the nuclear export of the large ribosomal subunit [29].

In this study, we investigate the role of Ns1 in proliferating epithelial tissues using the Drosophila wing imaginal disc as a model. We demonstrate that knocking down *Ns1* in wing discs leads to strong apoptosis. However, unlike the reported effect on overexpressed RPs, we did not observe nucleolar accumulation of endogenous RPs upon *Ns1* knockdown. Instead, proteomic analysis and the following experiments reveal that Ns1 deficiency reduces the protein levels of most RPs without affecting their transcription or proteasomal degradation. We further demonstrate that loss of Ns1 upregulates the Xrp1/Irbp18 complex, which subsequently induces apoptosis through activating JNK and suppresses translation through a mechanism independent of JNK and eIF2α phosphorylation.

## Results

### Loss of Ns1 induces apoptosis

**Fig. 1:**
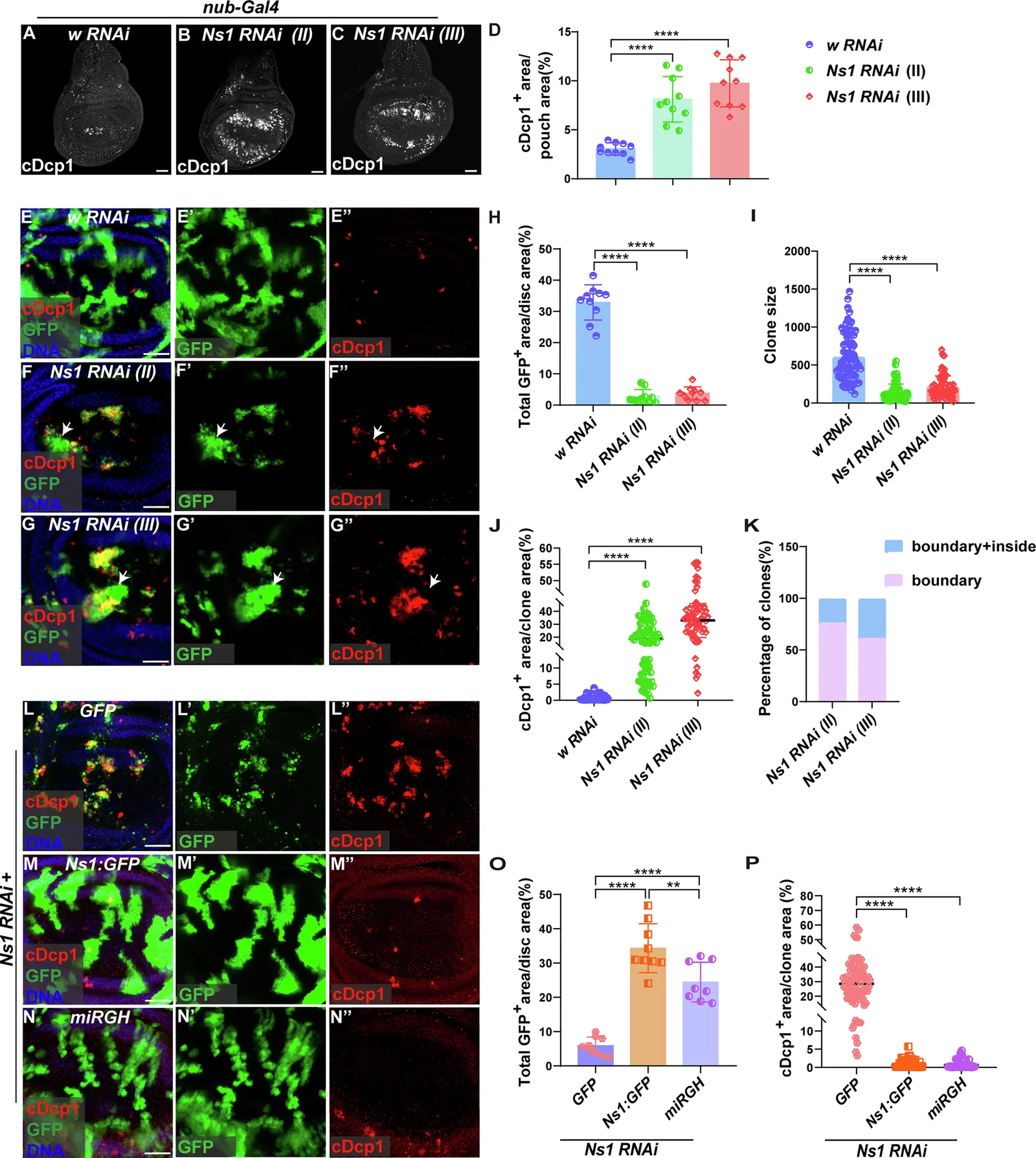
Loss of Ns1 induces apoptosis. **A**–**C** The representative images of cleaved Dcp-1 (cDcp-1) staining to show apoptosis in wing discs with the indicated transgenes expressed in pouch area under the control of *nub-Gal4*. Scale bars, 20 μm. **D** The percentage of cDcp1^+^ area in the pouch area of wing discs. *n* = 10 (*w RNAi*), 10 (*Ns1 RNAi II*), 10 (*Ns1 RNAi III*). **E**–**G** The representative images of cDcp-1 staining in GFP^+^ clones expressing the indicated transgenes. Scale bars, 20 μm. White arrows point to examples of big clones in which the interior and boundary cDcp1^+^ staining can be distinguished. **H** The percentage of GFP^+^ area in the disc area. n = 10 (*w RNAi*), 10 (*Ns1 RNAi II*), 10 (*Ns1 RNAi III*). **I** The sizes of individual clones. n = 77 clones from 13 discs (*w RNAi*), 72 clones from 15 discs (*Ns1 RNAi II*), 75 clones from 17 discs (*Ns1 RNAi III*). The sizes are presented in arbitrary units. **J** The fraction of cDcp1^+^ cells within the GFP^+^ clones. *n* = 79 clones from 13 discs (*w RNAi*), 71 clones from 15 discs (*Ns1 RNAi II*), 71 clones from 17 discs (*Ns1 RNAi III*). **K** The percentage of clones with cDcp1^+^ staining only at the boundary or both at the boundary and inside. *n* = 43 clones (*Ns1 RNAi II*) and 47 clones (*Ns1 RNAi III*) in total. **L**–**N** The representative images of cDcp-1 staining in GFP^+^ clones expressing the indicated transgenes. Scale bars, 20 μm. **O** The percentage of GFP^+^ area in the disc area. *n* = 9 (*Ns1 RNAi* + *GFP*), 9 (*Ns1 RNAi* + *Ns1: GFP*), 8 (*Ns1 RNAi +miRGH*). **P** The fraction of cDcp1^+^ cells within the GFP^+^ clones. *n* = 81 clones from 17 discs (*Ns1 RNAi* + *GFP*), 84 clones from 15 discs (*Ns1 RNAi* + *Ns1: GFP*), 70 clones from 9 discs (*Ns1 RNAi +mi RGH*). **P < 0.01. ****P < 0.0001. The genotypes of samples used in this figure are listed in Table S4.

Drosophila wing imaginal disc is a commonly used model to study epithelial homeostasis. To investigate the function of Ns1 in epithelial cells, we first knocked down *Ns1* in the pouch region of the wing disc by expressing two independent *Ns1 RNAi* under the control of *nub-Gal4* or *MS1096-Gal4*. Compared to control discs expressing RNAi targeting *white* (*w*) gene, expression of either *Ns1 RNAi* resulted in increased apoptosis, as detected by immunostaining for cleaved Dcp-1 (cDcp-1), an active form of Drosophila executioner caspase (Figs. 1A–D, S1A–S1C, E). Co-expression of Ns1:GFP together with *Ns1 RNAi* effectively suppressed the elevated apoptosis (Figs. S1D, S1E). These data suggest that depletion of Ns1 induces apoptosis.

We also generated clones expressing *Ns1 RNAi* or control RNAi in wing discs. Compared to control clones, *Ns1* knockdown clones showed a pronounced reduction in both number and size, accompanied by strong apoptosis (Fig. 1E–J). Among *Ns1* knockdown clones big enough to distinguish interior from boundary cDcp1 staining, more than 60% displayed apoptosis only at the clone boundary (Fig. 1K). This indicates that, besides autonomous apoptosis, depleting *Ns1* in clones may induce cell competition, which accelerates their elimination. The reduced number and size of *Ns1* knockdown clones were rescued by expression of exogenous Ns1:GFP, as well as by apoptosis inhibition through expression of miRGH, a microRNA targeting pro-apoptotic genes *reaper*, *hid* and *grim* [30] (Fig. 1L–P). Collectively, these findings underscore an essential role of Ns1 in cell survival.

### Ns1 deficiency results in a global reduction in RPs

Studies on mammalian NS have shown that its loss can activate p53, leading to cell cycle arrest and cell death [21, 22]. We therefore examined whether apoptosis induced by *Ns1* knockdown in wing discs is also mediated by p53 activation. Expression of a dominant-negative form of p53 (p53.DN) failed to suppress apoptosis in *Ns1* knockdown clones (Fig. S2), suggesting that loss of Ns1 induces apoptosis independent of p53.

**Fig. 2:**
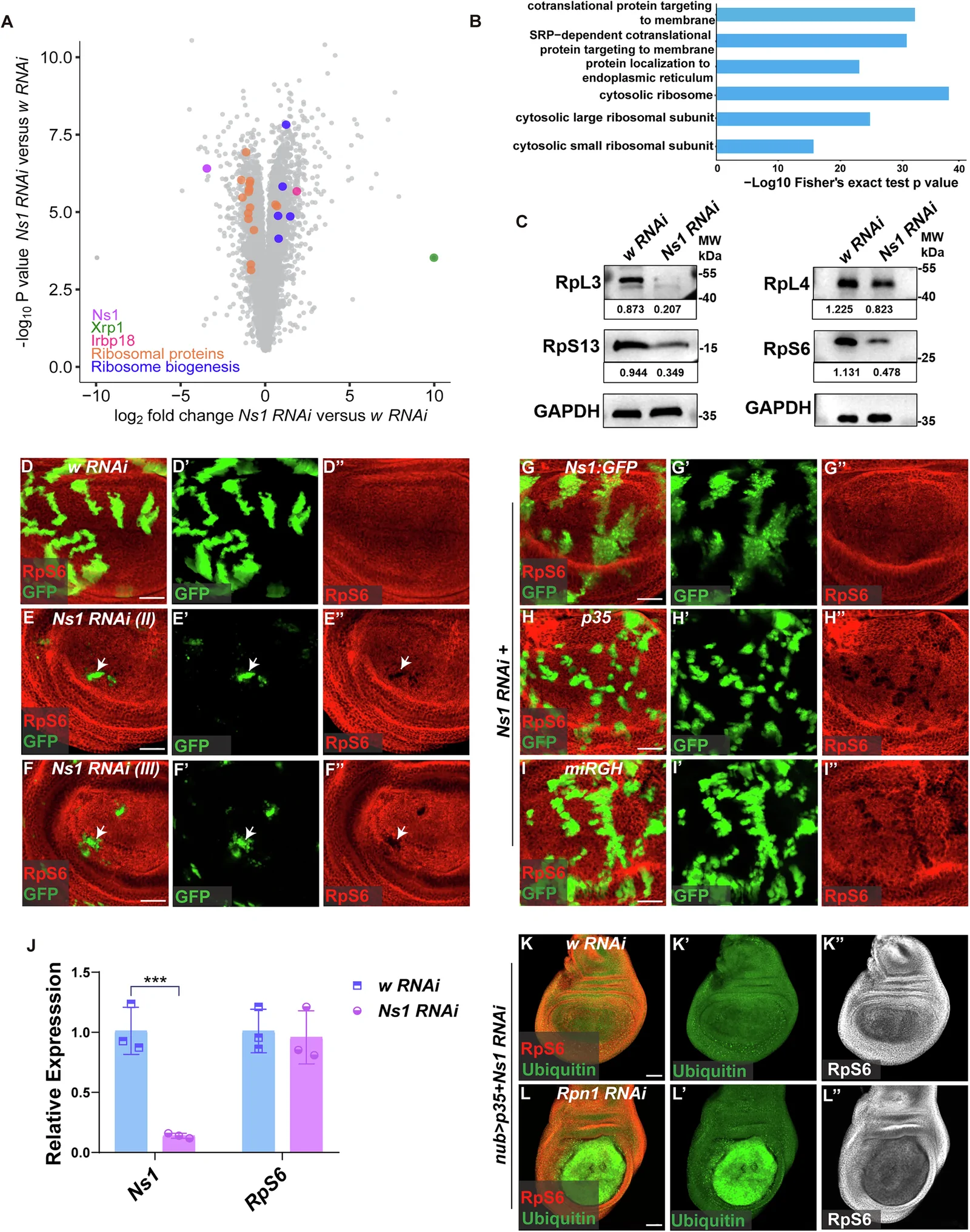
Loss of Ns1 results in reduction in RPs. **A** Volcano plot displaying differentially expressed proteins in *act* > *Ns1 RNAi* discs relative to *act* > *w RNAi*. The purple dot is Ns1. The green and red dots highlight Xrp1 and Irbp18, respectively. Blue dots are a few examples of proteins related to ribosome biogenesis. Orange dots are a few examples of RPs. **B** GO enrichment analysis of proteins downregulated in *act* > *Ns1 RNAi* discs. **C** Western blots showing the protein levels of RpL3, RpL4, RpS13 and RpS6 in *act* > *w RNAi* and *act* > *Ns1 RNAi* wing discs. The numbers below the blots are the ratios between the intensity of the band and that of the GAPDH band. Each protein sample was prepared from ~100 discs. **D**–**I** The representative images of RpS6 staining in clones (GFP^+^) expressing the indicated transgenes. The white arrows in (**E**) and (**F**) point to examples of small *Ns1* knockdown clones. Scale bars, 20 μm. **J** The relative mRNA levels of *Ns1* and *RpS6* in wing discs with *w RNAi* or *Ns1 RNAi* expressed in the whole wing discs using *act-Gal4*. The average mRNA level in *act* > *w RNAi* group was set as 1. *n* = 3. Each biological replicate was prepared from ~100 wing discs. ***: *P* < 0.001. **K**, **L** The representative images of RpS6 and ubiquitin staining in wing discs with the indicated transgenes expressed in the pouch under the control of *nub-Gal4*. Scale bar, 20 μm. The genotypes of samples used in this figure are listed in Table S4.

To elucidate the molecular mechanism by which Ns1 regulates apoptosis, we compared the proteome of *Ns1* knockdown wing discs with that of the control discs. 809 proteins were downregulated and 1148 were upregulated in *Ns1* knockdown discs (|Fold change | ≥1.5, *P* value < 0.05; Fig. 2A, Dataset S1). As expected, Ns1 protein itself was markedly downregulated in *Ns1* knockdown discs (Fig. 2A). Gene Ontology (GO) enrichment analysis revealed that the downregulated proteins were enriched in GO terms related to ribosomes and translation (Fig. 2B, Dataset S1). Interestingly, knockdown of *Ns1* caused a global reduction in RPs. Among the 79 RPs identified by mass spectrometry, 65 were downregulated (Table S1). We confirmed the downregulation of RpS6, RpS13, RpL3, and RpL4 in *Ns1* knockdown discs by Western blotting using commercially available antibodies (Fig. 2C). Immunostaining also revealed a dramatic reduction of RpS6 and RpS13 proteins in *Ns1* knockdown clones (Figs. 2D–F, S3A–C), a phenotype that was rescued by expression of exogenous Ns1 (Figs. 2G, S3D). To determine whether the reduction in RP levels is due to apoptosis, we inhibited apoptosis in *Ns1 RNAi* clones by expressing miRGH or p35, an inhibitor of apoptotic caspases [31, 32]. While blocking apoptosis increased the number and size of *Ns1 RNAi* clones, RpS6 and RpS13 remained downregulated (Figs. 2H, I, S3E, F). In addition, using a transgenic RpL3:Flag expressed under the control of the endogenous *RpL3* promoter, we confirmed the reduced RpL3 in *Ns1 RNAi* clones with or without apoptosis inhibition by immunostaining (Figs. S3G-S3J). These data together suggest that loss of Ns1 causes a reduction of RPs.

To investigate how Ns1 deficiency reduces RP levels, we first examined mRNA levels of some RPs and found that most were unaffected (Figs. 2J, S4), suggesting that Ns1 primarily regulates RPs at the post-transcriptional level. We then evaluated whether loss of Ns1 accelerates proteasomal degradation of RPs. We interfered with proteasome function by knocking down *Rpn1*, which encodes a proteasome subunit, in the wing pouch alongside *Ns1* knockdown and assessed the RpS6 level. To prevent severe cell loss due to apoptosis caused by the knockdown of *Rpn1* and *Ns1*, p35 was co-expressed. While expression of *Rpn1 RNAi* effectively disrupted proteasome function, as indicated by accumulation of ubiquitinated proteins, it did not restore the RpS6 level in *Ns1* knockdown tissues (Fig. 2K, L). This suggests that the reduced RP abundance in Ns1-deficient cells is not due to accelerated proteasomal degradation.

### Loss of Ns1 impairs rRNA processing and global translation

**Fig. 3:**
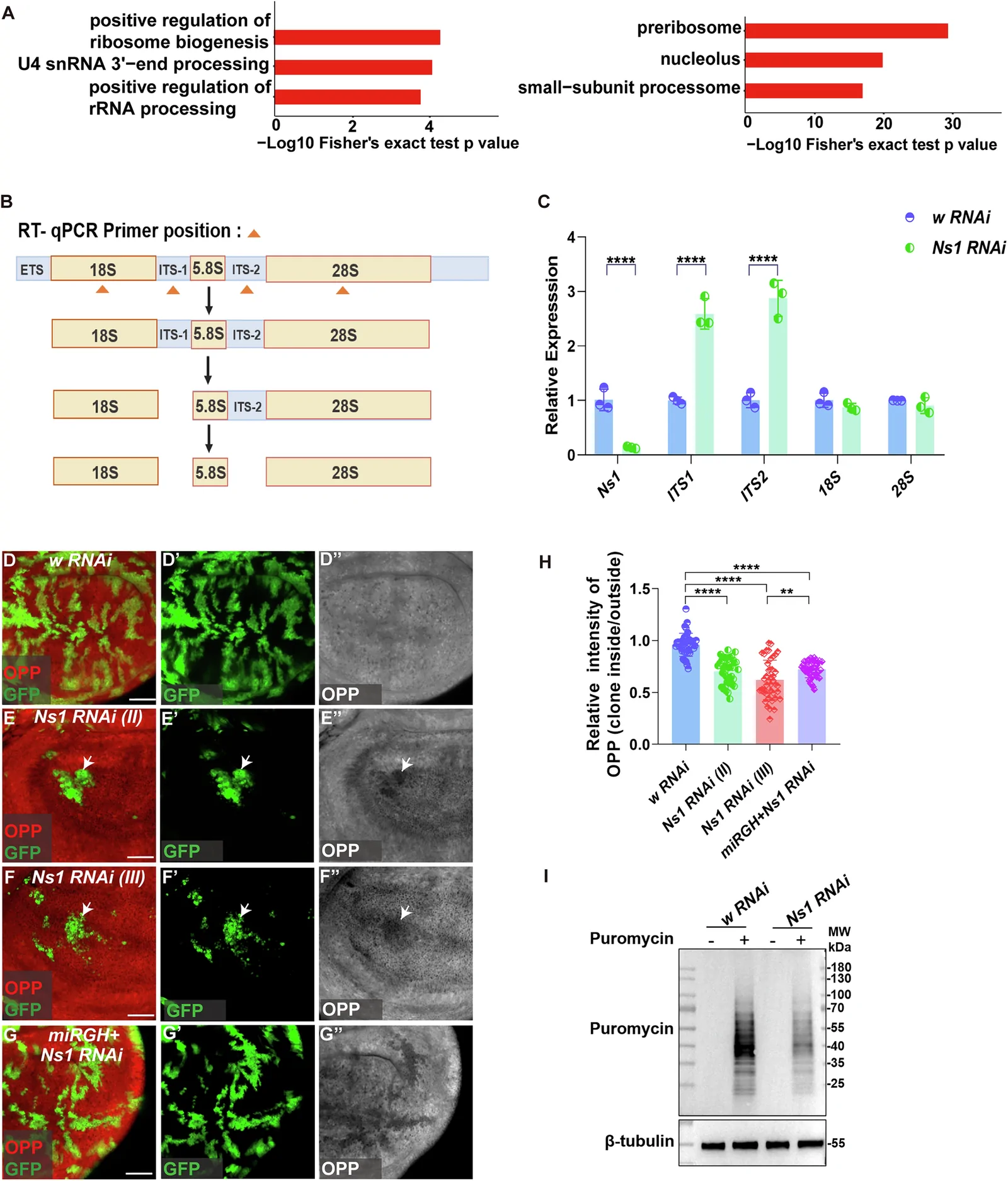
Ns1 deficiency impairs rRNA processing and translation. **A** GO enrichment analysis of proteins upregulated in *act* > *Ns1 RNAi* discs. **B** A schematic of rRNA processing. The orange triangles mark the positions where the qPCR primers match. **C** The relative rRNA levels in *act* > *w RNAi* and *act* > *Ns1 RNAi* wing discs detected by the indicated primers. Primers targeting ITS-1 and ITS-2 detect pre-rRNAs, and primers targeting 18S and 28S regions detect both pre-rRNA and mature rRNA. The average level in *act* > *w RNAi* group was set as 1. n = 3. Each biological replicate was prepared from ~100 wing discs. **D**–**G** The representative images showing OPP incorporation in clones (GFP^+^) expressing the indicated transgenes. Scale bars, 20 μm. The white arrows in (**E**) and (**F**) point to examples of *Ns1* knockdown clones. **H** The ratio between the average OPP intensity within the GFP^+^ clone and that in a region with comparable size outside the clones in the same disc. n = 40 clones from 8 discs (*w RNAi*), 40 clones from 17 discs (*Ns1 RNAi II*), 43 clones from 18 discs (*Ns1 RNAi III*), 42 clones from 18 discs (*Ns1 RNAi+ miRGH*). **I** Western blots showing puromycin incorporation levels in *nub* > *w RNAi* and *nub* > *Ns1 RNAi* wing discs. **P < 0.01; ****P < 0.0001. The genotypes of samples used in this figure are listed in Table S4.

GO analysis of proteins upregulated in *Ns1* knockdown discs revealed an enrichment of proteins involved in rRNA processing and ribosome biogenesis (Fig. 3A). To assess the effect of *Ns1* knockdown on rRNA processing, we quantified the levels of rRNA precursors using primers targeting internal transcribed spacer (ITS)-1 and ITS-2 (Fig. 3B). Reduction of Ns1 induced accumulation of rRNA precursors while total levels of mature rRNA and precursors, detected with primers targeting 18S and 28S regions, remained unchanged (Fig. 3B, C). These results together indicate impaired rRNA processing in *Ns1*-deficient tissues, consistent with previous reports on NS family proteins in other species [26–28]. The upregulation of proteins related to rRNA processing and ribosome biogenesis likely represents a compensatory response in Ns1-deficient tissues to counteract the impaired rRNA processing and reduced RPs.

Given that ribosomes are the translation machinery, the concurrent downregulation of numerous RPs and the disruption of rRNA processing in Ns1-deficient tissues prompted us to examine the effect on global translation. We assessed translation activity by incubating wing discs with *O*-propargyl puromycin (OPP) and visualizing puromycilated peptides in situ through a click-chemistry reaction. Reduced OPP incorporation was observed in *Ns1* knockdown clones, both in the presence and absence of apoptosis inhibition (Fig. 3D–H). The impaired translation was confirmed in wing discs with *Ns1* knocked down in the pouch region (Fig. 3I). Taken together, our results indicate that loss of Ns1 impairs rRNA processing, reduces RP levels and suppresses global protein synthesis.

### Ns1 deficiency triggers apoptosis and translation through upregulating the Xrp1/Irbp18 complex

The above findings demonstrate that loss of Ns1 causes reduced RP levels. Heterozygous mutations of individual RPs have been reported to cause unassembled RPs to aggregate, resulting in proteotoxic stress and apoptosis. Reducing translation by inhibiting the mTOR pathway can relieve the proteotoxic stress and prevent apoptosis [33, 34]. Thus, we asked whether inhibition of mTOR pathway could suppress apoptosis induced by loss of Ns1. Knocking down *Rheb* in wing discs strongly reduced phosphorylation of S6K and RpS6 and suppressed translation (Figs. S5A & S5B), indicating effective inhibition of mTOR signaling. However, this intervention did not affect apoptosis in Ns1-deficient clones (Figs. S5C–S5F), suggesting that, unlike the case with mutations in individual RPs, apoptosis triggered by Ns1 deficiency is not due to proteotoxic stress.

**Fig. 4:**
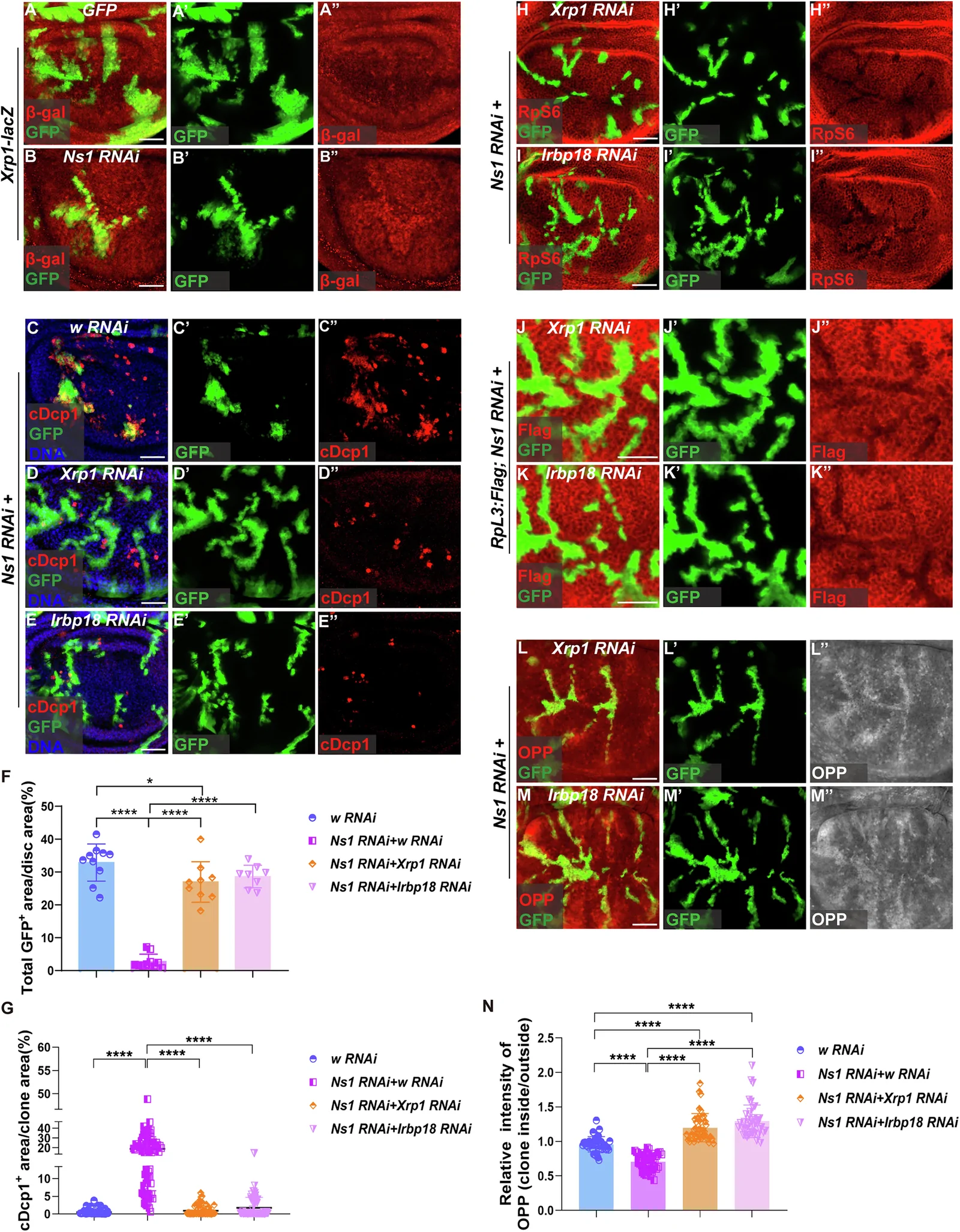
Ns1 deficiency triggers apoptosis through upregulating the Xrp1/Irbp18 complex. **A**, **B** The representative images of β-gal staining in discs carrying *Xrp1-lacZ* and with clones (GFP^+^) expressing the indicated transgenes. Scale bars, 20μm. **C**–**E** The representative images of cDcp1 staining in the clones (GFP^+^) expressing the indicated transgenes. Scale bars, 20 μm. **F** The percentage of GFP^+^ area in the disc area. n = 10 (*w RNAi*), 10 (*Ns1 RNAi* + *w RNAi*), 9 (*Ns1 RNAi+Xrp1 RNAi*), 8 (*Ns1 RNAi+ Irbp18 RNAi*). **G** The fraction of cDcp1^+^ cells within the GFP^+^ clones. n = 79 clones from 13 discs (*w RNAi*), 71 clones from 15 discs (*Ns1 RNAi* + *w RNAi*), 78 clones from 16 discs (*Ns1 RNAi+Xrp1 RNAi*), 78 clones from 10 discs (*Ns1 RNAi+ Irbp18 RNAi*). The representative images of RpS6 (**H**, **I**) or RpL3:Flag (**J**, **K**) staining in the clones (GFP^+^) expressing the indicated transgenes. Scale bar, 20 μm. **L**, **M** The representative images showing OPP incorporation in wing discs with the indicated transgenes expressed in GFP^+^ clones. Scale bars, 20 μm. **N** The ratio between the average OPP intensity within the GFP^+^ clone and that in a region with a comparable size outside the clones in the same disc. *n* = 40 clones from 8 discs (*w RNAi*), 40 clones from 17 discs (*Ns1 RNAi*), 41 clones from 16 discs (*Ns1 RNAi+ Xrp1 RNAi*), 41 clones from 12 discs (*Ns1 RNAi+ Irbp18 RNAi*). *P < 0.05; ****P < 0.0001. The genotypes of samples used in this figure are listed in Table S4.

Heterozygous *Rp* mutations have been reported to induce apoptosis and suppress translation through increasing the transcription factor Xrp1 [35, 36]. Our proteomic analysis revealed marked upregulation of Xrp1 and its binding partner Irbp18 in *Ns1* knockdown discs (Fig. 2A). This upregulation was further validated by RT-qPCR (Fig. S6A) and immunostaining of an *Xrp1-lacZ* reporter (Figs. 4A, B, S6B, C). To determine whether Ns1 deficiency induces apoptosis through upregulating the Xrp1/Irbp18 complex, we knocked down *Xrp1* or *Irbp18*. Knockdown of either gene suppressed apoptosis in Ns1-deficient tissues (Figs. 4C–G, S6D-H) without rescuing the downregulation of RpS6 and RpL3 (Fig. 4H–K), suggesting that Xrp1/Irbp18 acts downstream of RP reduction to regulate apoptosis. Notably, knockdown of *Xrp1* or *Irbp18* increased translation of *Ns1 RNAi* cells to a level slightly higher than that in the surrounding wild type cells (Figs. 4L–N, S6I), indicating that the Xrp1/Irbp18 complex plays an essential role in translation suppression under RP-deficient conditions. Taken together, these data indicate that the Xrp1/Irbp18 complex acts downstream of ribosomal defects to regulate apoptosis and translation in Ns1-deficient tissues.

### Loss of Ns1 triggers apoptosis by activating JNK

**Fig. 5:**
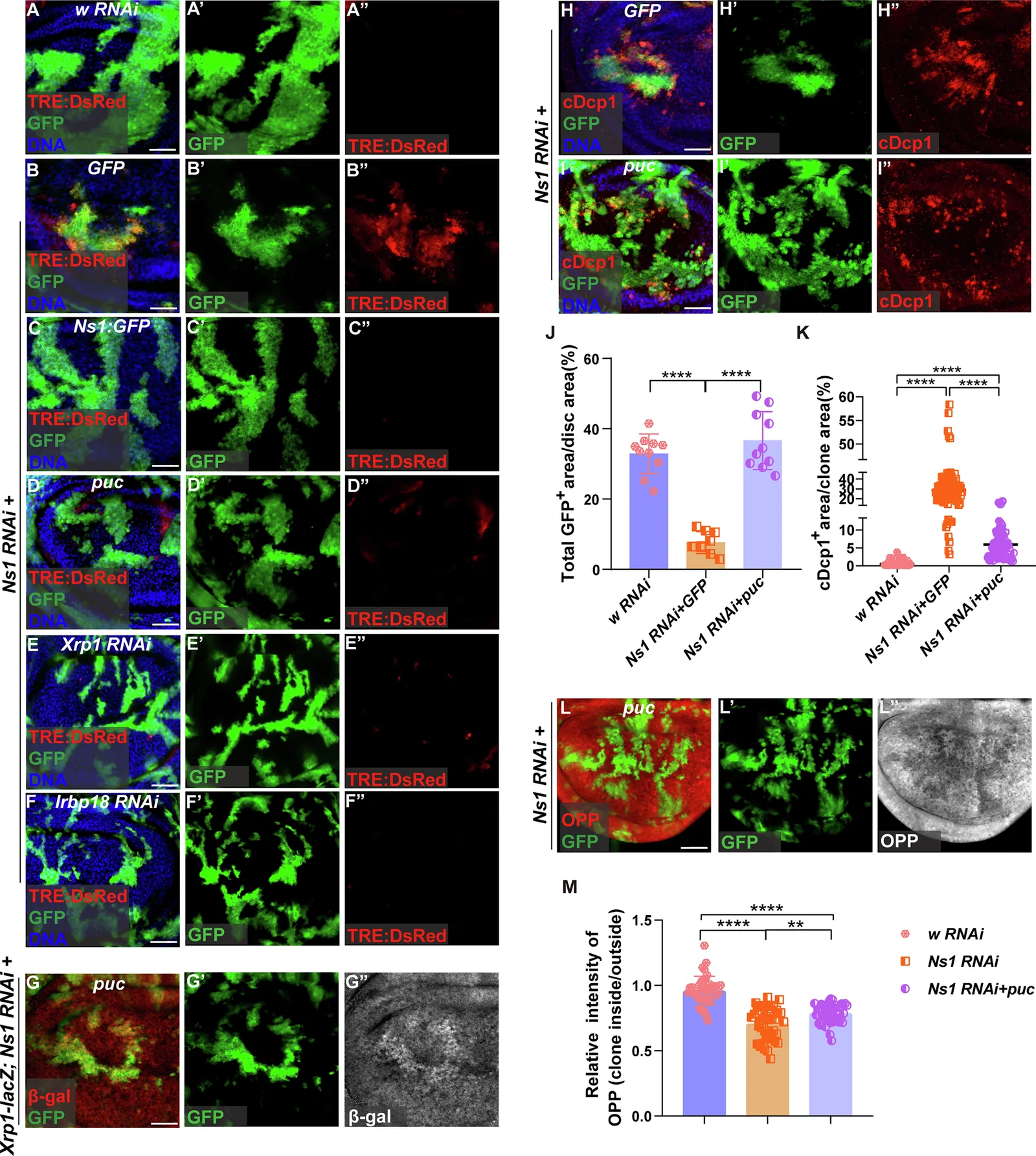
Ns1 deficiency activates JNK through upregulating the Xrp1/Irbp18 complex. **A**–**F** The representative images of TRE:DsRed in the clones (GFP^+^) expressing the indicated transgenes. **G** The representative images of *Xrp1-lacZ* staining in discs with clones (GFP^+^) co-expressing *Ns1 RNAi* and *puc*. Scale bar, 20 μm. **H**, **I** The representative images of cDcp1 staining in the clones (GFP^+^) expressing the indicated transgenes. Scale bar, 20 μm. **J** The percentage of GFP^+^ area in the disc area. n = 10 (*w RNAi*), 8 (*Ns1 RNAi* + *GFP*), 10 (*Ns1 RNAi+ puc*). **K** The fraction of cDcp1^+^ cells within the GFP^+^ clones. n = 79 clones from 13 discs (*w RNAi*), 82 clones from 17 discs (*Ns1 RNAi* + *GFP*), 81 clones from 15 discs (*Ns1 RNAi+ puc*). **L** The representative images showing OPP incorporation in discs with *Ns1 RNAi* and *puc* co-expressed in the GFP^+^ clones. Scale bars, 20 μm. **M** The ratio between the average OPP intensity within the GFP^+^ clone and that in a region with a comparable size outside the clones in the same disc. *n* = 40 clones from 8 discs (*w RNAi*), 40 clones from 17 discs (*Ns1 RNAi*), 40 clones from 9 discs (*Ns1 RNAi+ puc*). **P < 0.01; ****P < 0.0001. The genotypes of samples used in this figure are listed in Table S4.

Previous studies have demonstrated that Xrp1 can activate JNK to promote apoptosis [36–39]. To determine whether JNK mediates the regulation of apoptosis by Ns1, we utilized a JNK activity reporter, TRE:DsRed [40]. Knocking down *Ns1* upregulated TRE:DsRed expression, an effect that was suppressed either by expression of Ns1:GFP or by expression of puckered (puc), a negative regulator of JNK signaling (Fig. 5A–D). These data suggest that reduced Ns1 causes activation of the JNK pathway. Furthermore, knocking down *Xrp1* or *Irbp18* blocked JNK activation in Ns1-deficient clones (Fig. 5E, F), whereas inhibition of JNK did not affect Xrp1 expression (Fig. 5G). These results place the Xrp1/Irbp18 complex upstream of JNK activation upon loss of Ns1.

We next investigated whether Ns1 deficiency triggers apoptosis and suppresses translation through activation of JNK. Inhibition of JNK signaling through expression of *puc* restored the sizes of *Ns1* knockdown clones (Fig. 5H–J). Although some apoptotic cells persisted at the clone boundaries, inhibition of JNK markedly reduced apoptosis in *Ns1* knockdown clones (Fig. 5K), suggesting that Ns1 deficiency induces apoptosis through activating JNK. We further examined whether JNK is responsible for translation suppression caused by *Ns1* knockdown. OPP incorporation assays revealed that JNK inhibition failed to rescue the suppression of translation (Fig. 5L, M). These results indicate that JNK mediates apoptosis but not translation suppression caused by loss of Ns1.

### Loss of Ns1 induces translation suppression independent of eIF2α phosphorylation

**Fig. 6:**
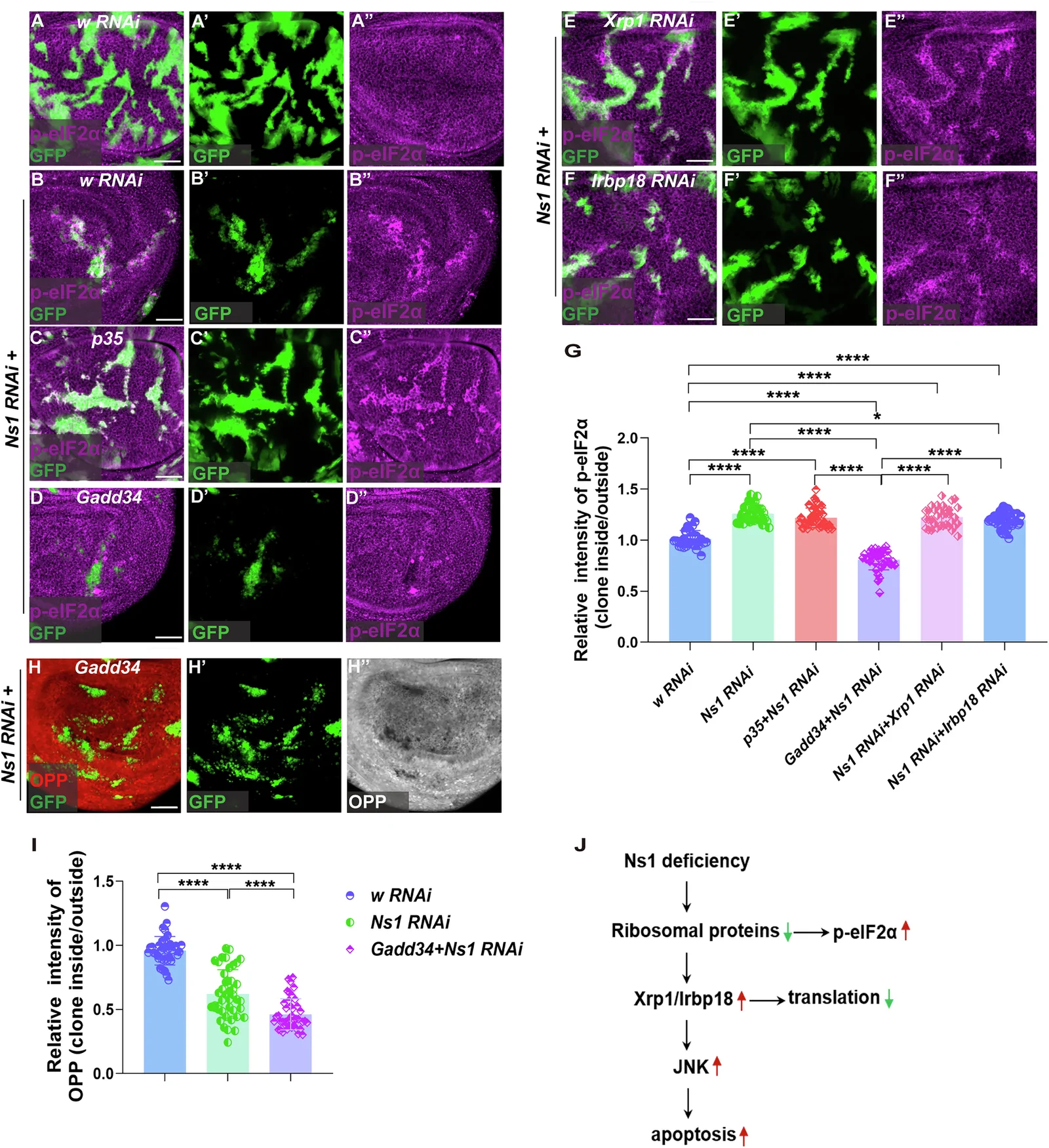
Ns1 deficiency suppresses translation independent of eIF2α phosphorylation. **A**–**F** The representative images of phosphorylated (p-) eIF2α staining in discs with clones (GFP^+^) expressing the indicated transgenes. Scale bars, 20 μm. **G** The ratio between the average p-eIF2α intensity within the clone and that in a region of comparable size outside of the clone in the same disc. n = 30 clones from 10 discs (*w RNAi*), 32 clones from 20 discs (*Ns1 RNAi* + *w RNAi*), 42 clones from 10 discs (*p35* + *Ns1 RNAi*), 36 clones from 17 discs (*Gadd34* + *Ns1 RNAi*), 37 clones from 16 discs (*Ns1 RNAi+ Xrp1 RNAi*), 45 clones from 19 discs (*Ns1 RNAi+ Irbp18 RNAi*). **H** The representative images showing OPP incorporation in discs with *Ns1 RNAi* and *Gadd34* co-expressed in the GFP^+^ clones. Scale bars, 20 μm. **I** The ratio between the average OPP intensity within the GFP^+^ clone and that in a region with a comparable size outside the clones in the same disc. *n* = 40 clones from 8 discs (*w RNAi*), 43 clones from 18 discs (*Ns1 RNAi*), 42 clones from 14 discs (*Ns1 RNAi+ Gadd34*). *P < 0.05; ****P < 0.0001. The genotypes of samples used in this figure are listed in Table S4. **J** A schematic demonstrating the mechanism by which Ns1 deficiency regulates apoptosis and translation.

The data above suggest that Ns1 deficiency suppresses translation through upregulation of the Xrp1/Irbp18 complex. Xrp1 has been reported to inhibit translation through phosphorylation of eIF2α [35, 41]. Consistently, we observed increased eIF2α phosphorylation in *Ns1* knockdown clones, which was blocked by overexpression of Gadd34, an eIF2α phosphatase, but not by apoptosis inhibition (Fig. 6A–D, G). Surprisingly, although knocking down *Xrp1* or *Irbp18* in Ns1-deficienct clones suppressed apoptosis (Fig. 4C-G) and restored translation (Fig. 4L–N), it did not prevent the elevated eIF2α phosphorylation (Fig. 6E–G). This indicates that loss of Ns1 induces eIF2α phosphorylation independent of the Xrp1/Irbp18 complex. Furthermore, *Gadd34* overexpression failed to restore translation or inhibit apoptosis in Ns1-deficient clones (Figs. 6H, I, S7), indicating that translation suppression and apoptosis induced by Ns1 deficiency occur independent of eIF2α phosphorylation.

## Discussion

In this study, we demonstrate that Drosophila Ns1, a homolog of human NS, plays an essential anti-apoptotic role in proliferating epithelial cells. Using the Drosophila wing disc as a model, we show that loss of Ns1 disrupts rRNA processing and broadly reduces RP levels. This ribosomal deficiency leads to upregulation of the Xrp1/Irbp18 complex, which in turn activates JNK to induce apoptosis and suppresses translation independent of JNK and eIF2α phosphorylation (Fig. 6J).

Studies on mammalian NS have demonstrated that its expression is high in proliferating cells and low in differentiated cells. Loss of NS in proliferating cells, either in stem/progenitor cells or in cancer cells, induces cell cycle arrest and cell death, primarily through activating p53 [21, 22, 42–44]. Both downregulation and overexpression of mammalian NS can activate p53. Excessive NS directly binds to MDM2 and inhibits MDM2-mediated p53 ubiquitylation and degradation, whereas reduced NS triggers nucleolar stress, leading to the association of RpL5 and RpL11 with MDM2 and subsequent inhibition of MDM2 function [21, 22]. In Drosophila wing discs, which consist of proliferating epithelial cells, we found that knocking down *Ns1* resulted in extensive apoptosis, indicating that Ns1 is essential for cell survival in this tissue. However, inhibition of p53 did not suppress apoptosis in Ns1-deficient cells, suggesting that the mechanisms underlying apoptosis induced by loss of NS family proteins are context-dependent.

The nucleolus is the primary site for ribosome biogenesis. NS family proteins, which localize to the nucleolus, have been implicated in the regulation of rRNA processing. In HeLa cells, immunoprecipitation experiments by Romanova et al. demonstrated that human NS interacts with proteins involved in rRNA processing, such as DDX21, Pes1, and EBP2. Depletion of NS impairs, while its overexpression facilitates, the processing of 32S pre-rRNA into 28S rRNA [28]. Subsequently, Lin et al. reported that knockdown of *GNL3L*, a paralog of *NS* in human cells, induces a more rapid and severe impairment in rRNA processing and ribosome production compared to *NS* knockdown [45]. Studies in C. elegans and zebrafish further support a role for NS family proteins in rRNA processing [26, 27]. Consistent with these findings, our work in Drosophila wing discs shows that loss of Ns1 leads to accumulation of rRNA precursors, underscoring the conserved function of NS family proteins in this process. However, analyses of NS localization in *C. elegans* and mouse cells reveal that NS is not enriched in sub-nucleolar regions where the transcription and processing of rRNA occur, and its distribution differs from that of 28S rRNA [26, 46]. Therefore, whether the effect of NS loss on rRNA processing is direct or indirect remains unclear.

In addition to impaired rRNA processing, our proteomic analysis revealed, unexpectedly, that loss of Ns1 resulted in reduced levels of 65 out of 79 RPs in wing discs, suggesting an essential role for Ns1 in maintaining global RP abundance. The impact of NS family protein depletion on RP levels has not been studied in other species. Given that different steps in ribosome biogenesis are highly coordinated, the reduced rRNA processing in Ns1-deficient cells could be indirectly triggered by the global reduction of RPs. Previously, Rosby et al. overexpressed fluorescence-tagged RpL11 and RpL26 in Drosophila midgut polyploid epithelial cells and observed their nucleolar accumulation upon Ns1 depletion [29]. In contrast, we did not detect nucleolar accumulation of endogenous RPs in Ns1-deficient discs, possibly due to their inherently low abundance in these tissues. Although protein levels of most RPs decreased following loss of Ns1, mRNA levels of many RPs remained unchanged, suggesting Ns1 may not regulate transcription of RPs. While RPs are typically degraded via the ubiquitin-proteasome system [47, 48], proteasome inhibition did not restore RP levels in Ns1-deficient tissues, suggesting that loss of Ns1 does not enhance proteasomal degradation of RPs. The mechanism by which Ns1 regulates RP levels thus requires further investigation. One possibility is that loss of Ns1 suppresses translation of RPs. Most RPs and ribosome biogenesis factors share some common translational regulatory mechanisms, allowing ribosome abundance to adapt rapidly to extrinsic or intrinsic cues [49, 50]. However, given that Ns1 is a nuclear protein and translation occurs in the cytoplasm, any effect of Ns1 on RP translation is likely indirect, potentially mediated through factors such as small RNAs.

Mutations in a single RP can lead to ribosomopathies in humans. In Drosophila, heterozygous mutants of individual RPs have been extensively used to study cell competition, as heterozygous cells are eliminated through apoptosis when confronted with wild-type cells [51]. Even in non-competitive settings, apoptosis still occurs, albeit less severely, when an entire disc or compartment carries heterozygous RP mutations. Previous studies indicate that heterozygous RP mutations disrupt the stoichiometric balance between large subunits and small subunits, leading to aggregation of unassembled RPs and subsequent proteotoxic stress. Inhibiting the mTOR pathway, which reduces global translation, can alleviate apoptosis in these heterozygous RP mutant cells [33, 34]. In our study, although loss of Ns1 induced phosphorylation of eIF2α, which regulates CAP-dependent translation in response to proteotoxic stress, mTOR inhibition did not reduce apoptosis in Ns1-deficient discs. This indicates that loss of Ns1 may cause a balanced reduction in RPs and thus does not induce proteotoxic stress, and that the increased eIF2α phosphorylation may arise through other mechanisms. Alternatively, even if proteotoxic stress is induced in Ns1-deficient cells, it is not responsible for apoptosis in these cells.

In mammals, impaired ribosome biogenesis can activate p53, leading to cell cycle arrest and apoptosis [52]. Similarly, in Drosophila, defective ribosome biogenesis has been shown to activate p53 in germline cells and insulin-producing cells [53, 54]. In wing discs, knocking down *Noc1*, which reduces rRNA processing, also results in p53 activation and apoptosis [55–57]. In contrast, although loss of Ns1 disrupted ribosome biogenesis, apoptosis triggered by Ns1 deficiency was p53-independent and instead required the Xrp1/Irbp18 complex. This heterodimeric bZIP transcription factor complex is upregulated in cells with heterozygous RP mutations or upon depletion of translation factors such as eIF4G. Loss of Xrp1 or Irbp18 suppresses apoptosis in discs carrying RP mutations or lacking certain translation factors, indicating that this complex triggers apoptosis to eliminate translation-defective cells [35, 36, 58, 59]. In Ns1-deficient tissues, we detected upregulation of both Xrp1 and Irbp18. Importantly, knockdown of either factor suppressed apoptosis induced by loss of Ns1, supporting the conclusion that impaired ribosome biogenesis in these cells activates apoptosis through the Xrp1/Irbp18 complex. Although Xrp1 has been identified as a p53 target gene [60], the differential requirement for Xrp1 and p53 in Ns1-deficient cells indicates that Xrp1 upregulation in these cells does not rely on p53. Previous reports show RpS12 upregulates Xrp1 in cell competition triggered by mutations in other RPs [35, 37]. However, the proteomic analysis revealed reduced RpS12 in *Ns1* knockdown discs, suggesting that RpS12 is unlikely to mediate Xrp1 upregulation in this context. Further studies will be needed to elucidate how Ns1 regulates Xrp1.

The Xrp1/Irbp18 complex is known to mediate translation suppression in RP mutant cells [35, 36]. Consistent with this, we found that knockdown of *Xrp1* or *Irbp18* restored overall translation in Ns1-deficient clones. Notably, however, protein levels of RpS6 and RpL3 remained low, indicating that disrupting the Xrp1/Irbp18 complex does not restore RP levels. Previous studies show that loss of Xrp1 has little effect on rRNA processing and transcription of RP genes in RP mutants [35, 36], suggesting that translational recovery upon depletion of the Xrp1/Irbp18 complex may not result from increased ribosome biogenesis. It has been reported that Xrp1 induces phosphorylation of eIF2α to suppress translation in clones carrying mutations in individual *Rp* gene or in *mahjong* gene [36, 38, 41]. We also observed increased eIF2α phosphorylation in *Ns1* knockdown clones. However, knocking down *Xrp1* or *Irbp18* in these clones did not reduce eIF2α phosphorylation, implying that eIF2α phosphorylation in Ns1-deficient clones is induced via an Xrp1/Irbp18-independent mechanism. In addition, inhibition of eIF2α phosphorylation failed to restore overall translation or prevent apoptosis in Ns1-deficient clones. These findings indicate the existence of an eIF2α-independent pathway through which the Xrp1/Irbp18 suppresses translation. Further efforts will be required to elucidate the underlying mechanisms.

## Materials and methods

### Fly strains

*UAS-Ns1 RNAi* (BDSC# 29622) [61], *UAS-Ns1 RNAi* (BDSC# 61950) [61], *UAS-w RNAi* (BDSC# 33623) [61], *Xrp1-lacZ* (BDSC# 11569) [62], *UAS-p35* (BDSC# 5072), *tub-Gal80*^*ts*^ (BDSC# 7018) [63], *RpL3:3×Flag* (BDSC# 79221) [64], *TRE:DsRed* (BDSC# 59012) [40], *UAS-puc* (BDSC# 98328) [65] and *UAS-p53.DN* (BDSC# 8421) [66] were obtained from Bloomington Drosophila Stock Center (BDSC). *UAS-Xrp1 RNAi* (THU0591) [67], *UAS-Irbp18 RNAi* (THU0595) [67], *UAS-Rheb RNAi* (THU1308) [67], *UAS-Rpn1 RNAi* (THU1562) [67], *MS1096-Gal4* (TB00126) and *nub-Gal4* (THU00045) [68] were obtained from TsingHua Fly Center (Beijing, China). *hsFlp; ex-lacZ/CyO; act* > *CD2>Gal4 UAS-GFP* (BCF# 468) was obtained from the National Drosophila Resource Center of China (NDRCC). *UAS-miRGH* [30] was generously shared by Dr. David Bilder.

To generate *UAS-Ns1:GFP* transgenic flies, the coding sequence of *Ns1* was amplified using cDNA of wing discs as a template, and inserted into *pJFRC7-20×UAS-IVS-mCD8:GFP* (Addgene# 26220) between XhoI and BamHI using In-Fusion Cloning Kit (TakaraBio, Cat# 639650). The PCR primers used for amplifying Ns1 are listed in Table S2. The *pJFRC7-20×UAS-Ns1:GFP* plasmid was then injected into the attP40 site by Qidong Fangjing Biotechnology (Beijing, China).

### Fly experiments

To generate mosaic clones, the animals were raised at 25 °C for 3 days after egg laying, heat shock at 37 °C for 30 min and returned to 25 °C for an additional 2.5 days. To get wing discs with *Ns1 RNAi* ubiquitously expressed, *act* > *Ns1 RNAi tubGal80*^*ts*^ larva and the control *act* > *w RNAi tubGal80*^*ts*^ larva were raised at 18 °C for 4 days and then shifted to 29 °C for 2.5 days. Larva used in other experiments grew at 25 °C.

### Immunofluorescence staining and image analysis

The anterior part of the third instar larva was inverted in PBS and fixed in 4% cold paraformaldehyde (Sangon Biotech, Cat# E672002) for 20 min at room temperature. Samples were permeabilized in PBT (0.3% Triton X-100 in PBS) for 20 min followed by blocking with 5% goat serum in PBT for 30 min at room temperature. Samples were then incubated with primary antibodies diluted in blocking buffer overnight at 4 °C. The next day, after three times of 10-min wash with PBT, samples were incubated with secondary antibodies and Hoechst solution (Molecular Probes, Cat#33342) for 1 h in the dark at room temperature. Samples were then washed three times with PBT. Wing discs were dissected out and mounted on slides. Images were acquired using a two-photon laser confocal microscope (ZEISS LSM980). The software used in image capture was ZEN Black (Zeiss). The primary and secondary antibodies used in immunostaining are listed in Table S3.

All the image analyses and quantification were done using Fiji software. To quantify apoptosis and clone size, cDcp1 positive area, pouch or disc area and clone area were measured, and the ratio between them was calculated. Confocal planes covering all apoptotic cells (Dcp1^+^) and clones (GFP^+^) along the apicobasal axis of wing discs were maximum-projected using Fiji. The pouch was outlined manually, and its area was measured. To segment the apoptotic cells and clones, a threshold excluding the background was applied to generate a binary mask. The resulting regions of interest (ROIs) were used to determine the area covered by apoptotic cells or GFP^+^ clones. To quantify p-eIF2α and OPP staining, the average intensity within each GFP^+^ clone and in a GFP^-^ region of comparable area in the same disc was measured. The ratio of the intensity between the clone and the GFP^-^ region was then calculated.

### Proteomic analysis

Wing discs were dissected in cold PBS, snap-frozen, and stored at −80 °C. 300 wing discs were included per biological replicate, and three biological replicates for each group were prepared. After collecting enough discs, samples were lysed in extraction buffer containing 1% protease inhibitors, centrifuged at 12,000 × *g* for 10 min at 4 °C. The supernatant was then transferred to a new tube, and the protein concentration was determined using a BCA kit. For digestion, the protein solution was reduced with 5 mM dithiothreitol for 30 min at 56 °C and alkylated with 11 mM iodoacetamide for 15 min at room temperature in the dark. The protein sample was then diluted by adding 200 mM TEAB to a urea concentration less than 2 M. Finally, trypsin was added at a 1:50 trypsin-to-protein mass ratio for the first digestion overnight and a 1:100 trypsin-to-protein mass ratio for a second 4 h-digestion. Finally, the peptides were desalted by a Strata X SPE column.

The tryptic peptides were dissolved in solvent A (0.1% formic acid, 2% acetonitrile/in water), directly loaded onto a home-made reversed-phase analytical column (25-cm length, 100 μm i.d.). The mobile phase consisted of solvent A and solvent B (0.1% formic acid, 90% acetonitrile/in water). Peptides were separated with the following gradient: 0–22.5 min, 6–22% B; 22.5–26.5 min, 22–34% B; 26.5–28.5 min, 34–80% B; 28.5–30 min, 80% B, and all at a constant flow rate of 700 nl/min on an EASY-nLC 1200 UPLC system (ThermoFisher Scientific). The separated peptides were analyzed in Orbitrap Exploris 480 with a nano-electrospray ion source. The electrospray voltage applied was 2300 V. FAIMS compensate voltage (CV) was set as −45 V. Precursors and fragments were analyzed at the Orbitrap detector. The full MS scan resolution was set to 30000 for a scan range of 450–850 m/z. The MS/MS scan resolution was set to 30000. The HCD fragmentation was performed at a normalized collision energy (NCE) of 28%. The automatic gain control (AGC) target was set at 3E6, with a maximum injection time of Auto.

The DIA data were processed using the DIA-NN search engine (v.1.8). Tandem mass spectra were searched against the Drosophila_melanogaster_7227_PR_20250717.fasta (21999 entries) concatenated with a reverse decoy database. Trypsin /P was specified as the cleavage enzyme, allowing up to 1 missing cleavage. Excision on N-term Met and carbamidomethyl on Cys were specified as fixed modifications. FDR was adjusted to < 1%.

### Western blotting

Wing discs were lysed in RIPA lysis buffer (50 mM Tris, pH 7.4, 150 mM NaCl, 1% Triton X-100, 1% sodium deoxycholate, 0.1% SDS, sodium orthovanadate, sodium fluoride, EDTA, leupeptin protease inhibitor cocktail). The cell lysates were separated by SDS-PAGE and subsequently transferred onto PVDF membranes (Cobetter, Cat# 3550YH-R2703). After blocking with 5% non-fat powdered milk (Sangon Biotech, Cat# A600669), membranes were first probed with primary antibodies at 4°C overnight, followed by incubation with appropriate HRP-conjugated secondary antibodies for 1 hour at RT. Proteins were detected using Chemiluminescent Substrates (Spark Jade, Cat# ED0016) and Tanon 5200 Multi Chemiluminescence imager (Tanon Science & Technology Co., Shanghai, China). The antibodies used in Western blotting are listed in Table S3.

### Puromycin incorporation assay

To detect puromycin incorporation by Western blotting, the anterior parts of third-instar larva were inverted in Schneider’s medium and then transferred to Eppendorf tubes containing medium plus 5 μg/mL puromycin for 30 min incubation at room temperature. Following incubation, the inverted larvae were snap frozen to stop the incorporation. The wing discs were then isolated in cold PBS and lysed for Western blot analyses.

To visualize protein synthesis in situ, puromycin incorporation was assayed using the Click-iT Plus OPP Alexa Fluor Protein Synthesis Assay Kit (Thermo Fisher Scientific, Cat# C10457) according to the manufacturer’s protocol with modifications. The inverted anterior parts of larva were incubated in prewarmed Schneider’s medium with 20 µM OPP at room temperature for 30 min. After OPP incorporation, inverted larvae were washed with PBS twice and fixed in 4% formaldehyde and rinsed again in PBS before a permeabilization and blocking step. The Click-iT reaction mix was prepared according to the manufacturer’s instructions, and larvae were incubated for 30 min in the dark at room temperature. The samples were finally rinsed in reaction rinse buffer and stained with DAPI. Discs were dissected out and mounted in mounting medium.

### Reverse transcription-quantitative real time PCR (RT-qPCR)

RNA extraction was performed using VAMNE Magnetic Cell/Tissue Total RNA Kit (Vazyme, Cat# RMA101-C2-P1) following the manual. Extracted RNA was purified and reverse transcribed to cDNA using HiScript III RT SuperMix for qPCR (Vazyme, Cat# R323-01). qPCR reactions were performed with 1000 ng of cDNA template using ChamQ SYBR Color qPCR Master Mix (Vazyme, Cat# Q411-02) on Analytikjena Qtower. The primers used are listed in Table S2. The level of the RNA of interest was first normalized to that of *Act5C* mRNA in the same sample. This normalized value was then compared across different groups, with the average value from the control samples set as 1.

### Statistical analysis

Data are presented as the mean ± standard deviation (SD). Statistical analyses were performed using GraphPad Prism version 8 (GraphPad Software). Statistical significance was determined using a t-test for two-sample comparison or one-way ANOVA with Tukey test for comparing three or more samples. *P* < 0.05 was considered statistically significant. The sample sizes were chosen empirically based on the observed effects and previous reports. The sample size for each experiment is listed in the figure legends. The animals were grouped based on genotypes. When collecting data from qPCR and performing quantification on images, the investigators were blinded to the group allocation.

## Supplementary information

Supplementary figures and tables

Dataset S1

Uncropped Western blots

## Data Availability

The raw data of proteomic analysis can be accessed at ProteomeXchange with access number PXD075076 (http://proteomecentral.proteomexchange.org/cgi/GetDataset?ID=PXD075076). The original images of Western blots are provided as a supplementary document. All the other raw data supporting the conclusions, fly stocks and plasmids are available from the corresponding author upon request.
