# Supplementary material for: Loss of Drosophila nucleostemin 1 disrupts ribosomal protein homeostasis and rRNA processing to trigger apoptosis via the Xrp1/Irbp18 complex
Source: Cell Death Discov. 2026 Jun 14;12:342. doi: 10.1038/s41420-026-03205-9 (PMC13490407; doi:10.1038/s41420-026-03205-9)
Supplement: Supplementary file 1 [file 41420_2026_3205_MOESM1_ESM.pdf]

Supplementary information

Liu et al.

This file contains the following contents.

Fig S1-S7

Table S1-S4

The legend for Dataset S1

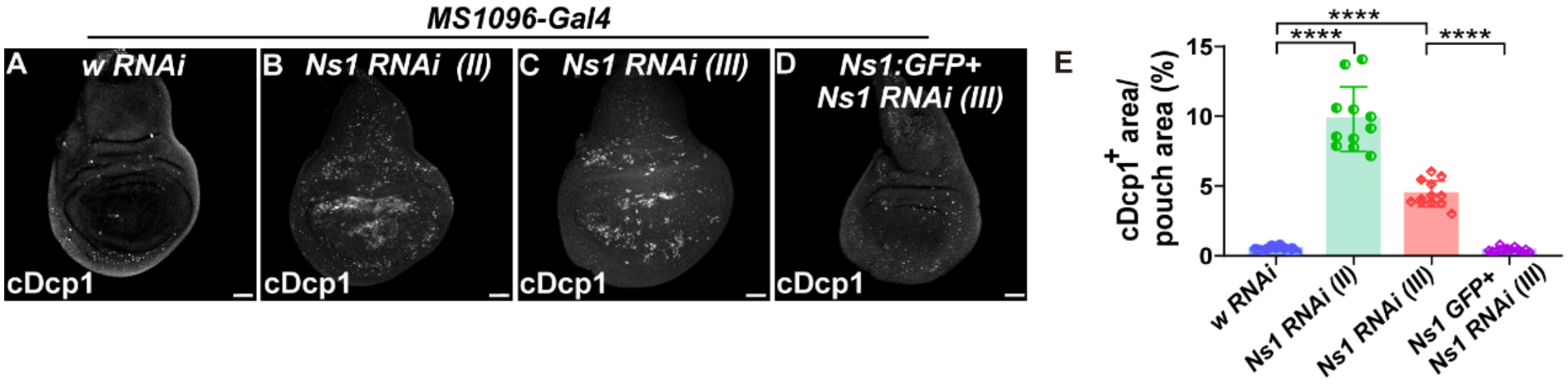

Fig S1. Knockdown of *Ns1* in wing pouch induces apoptosis.

(**A-D**) The representative images of cleaved Dcp-1 (cDcp-1) staining to show apoptosis in wing discs with the indicated transgenes expressed in pouch area under the control of *MS1096-Gal4*. Scale bars, 20 µm. (**E**) The percentage of $cDcp1^{+}$ area in the pouch area of wing discs. n=11 (*w RNAi*), 11 (*Ns1 RNAi II*), 12 (*Ns1 RNAi III*), 12 (*Ns1: GFP+ Ns1 RNAi*). ****: *P*< 0.0001. The genotypes of samples used in this figure are listed in Table S4.

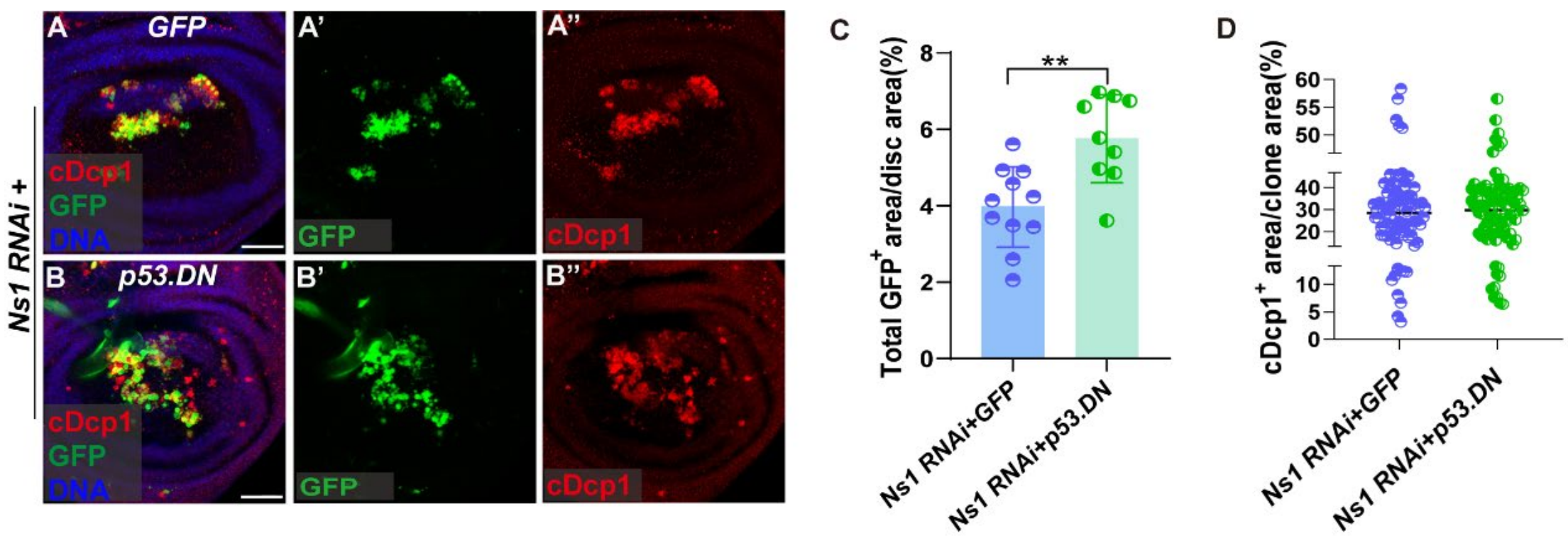

Fig S2. Inhibition of p53 does not suppress apoptosis induced by loss of Ns1.

(**A-B**) The representative images of GFP^{+} clones expressing the indicated transgenes. Scale bar, 20 μm. (**C**) The percentage of GFP^{+} area in the disc area. n= 11 (*Ns1 RNAi+ GFP*), 9 (*Ns1 RNAi+ p53.DN*). (**D**) The fraction of cDcp1^{+} cells within the GFP^{+} clones. n=81 clones from 17 discs (*Ns1 RNAi+ GFP*), 85 clones from 12 discs (*Ns1 RNAi+ p53.DN*). **: *P*< 0.01. The genotypes of samples used in this figure are listed in Table S4.

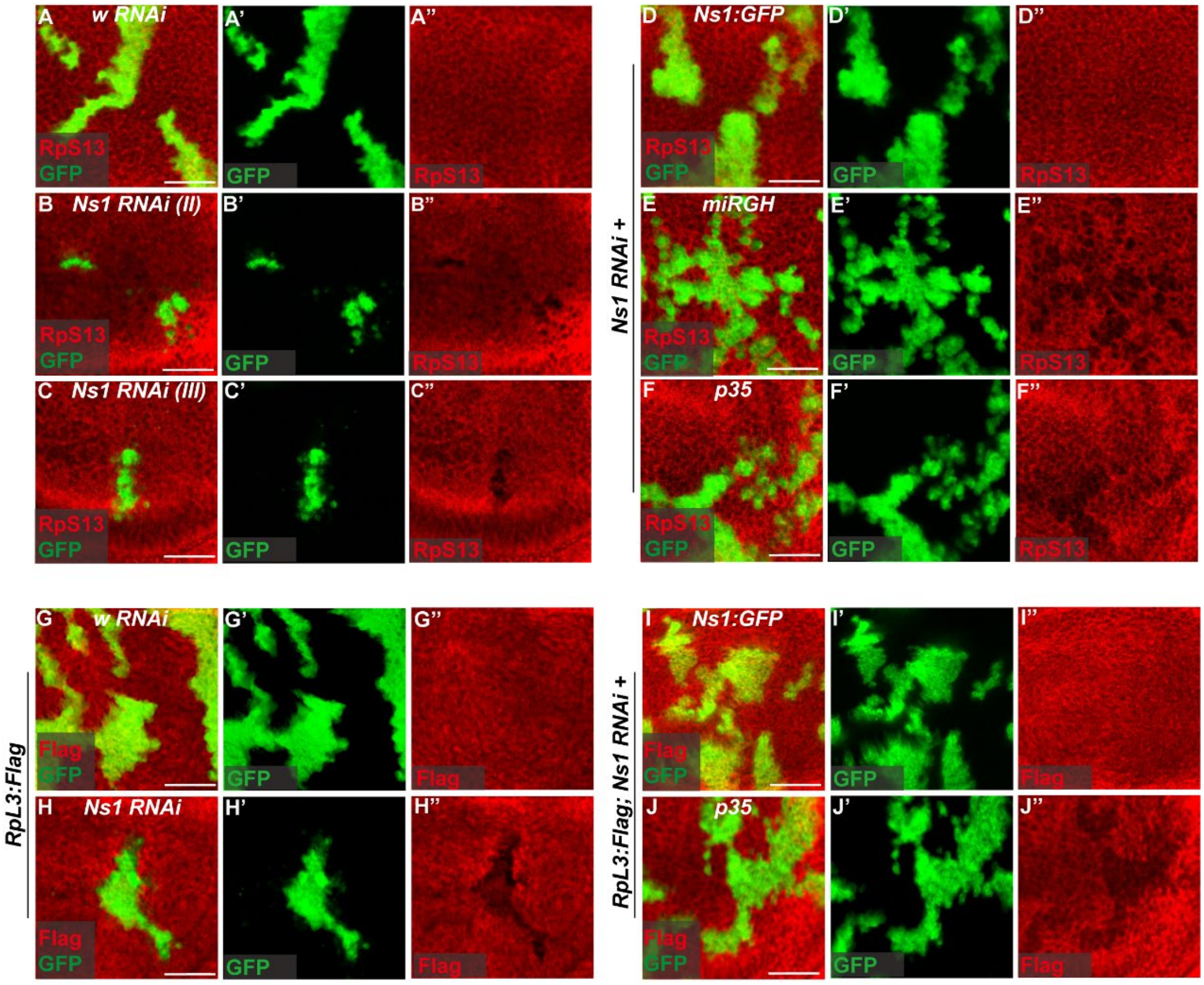

Fig S3. RpS13 and RpL3 are downregulated in *Ns1* knockdown clones.

(**A-F**) The representative images of RpS13 staining in clones (GFP^+) expressing the indicated transgenes. Scale bars, 20 µm. (**G-J**) The representative images of RpL3: Flag staining in clones (GFP^+) expressing the indicated transgenes. Scale bars, 20 µm. The genotypes of samples used in this figure are listed in Table S4.

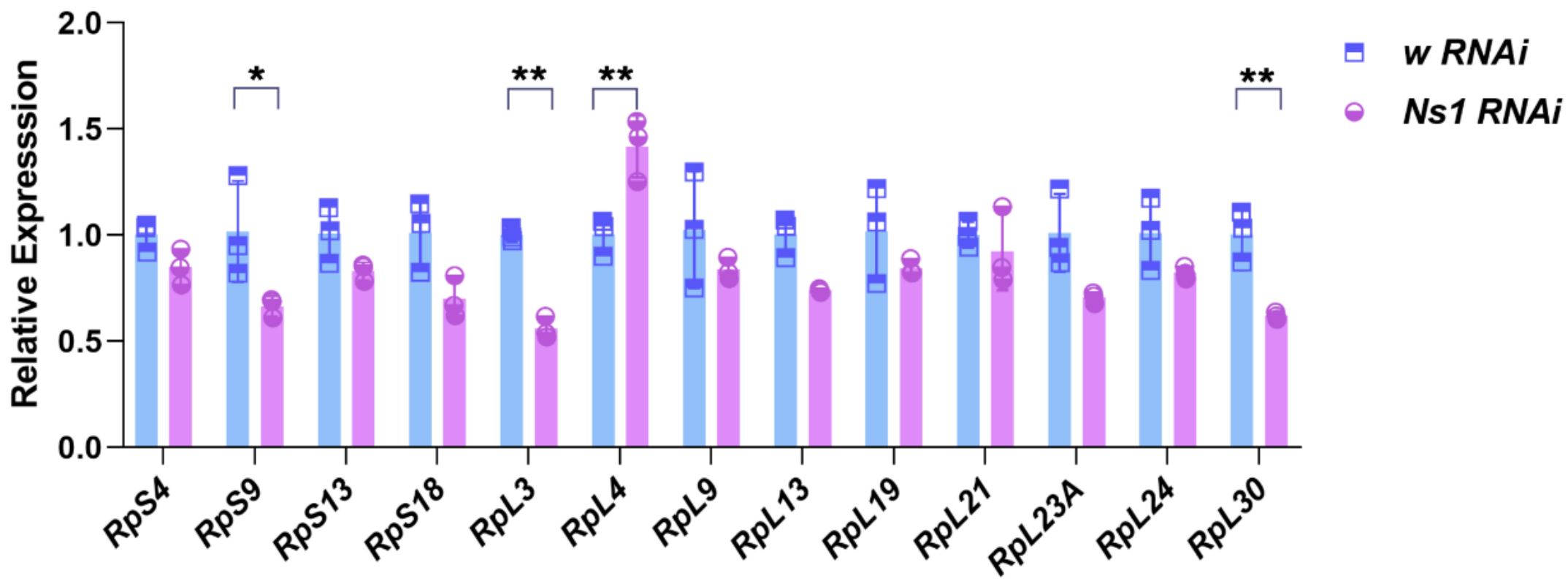

Fig S4. Ns1 deficiency does not affect the mRNA levels of RPs.

The relative mRNA levels of the indicated RPs in wing discs with *w RNAi* or *Ns1 RNAi* expressed in the whole wing discs using *act-Gal4*. The average mRNA level in *act*> *w RNAi* group was set as 1. n= 3. Each biological replicate was prepared from ~100 wing discs. *: *P*< 0.05; **: *P*< 0.01. The genotypes of samples used in this figure are listed in Table S4.

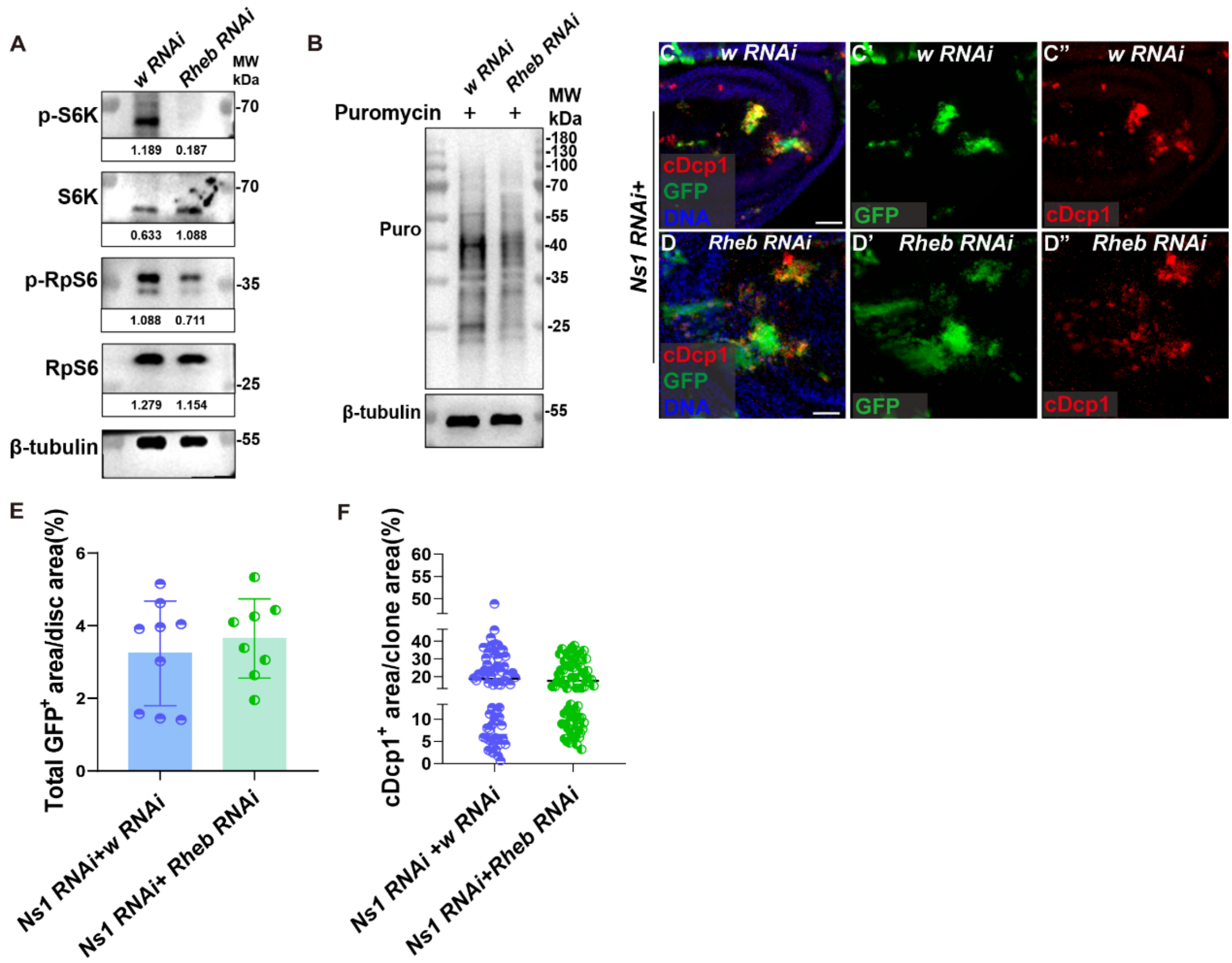

Fig S5. Inhibition of mTOR pathway fails to suppress apoptosis induced by Ns1 deficiency.

(**A**) Western blots showing the protein levels of phosphorylated (p-) S6K, S6K, p-RpS6 and RpS6 in *act>w RNAi* and *act>Rheb RNAi* wing discs. The numbers below the blots are the ratios between the intensity of the band and that of the tubulin band. Each protein sample was prepared from ~100 discs. (**B**) The blots showing puromycin incorporation in *act>w RNAi* and *act>Rheb RNAi* wing discs. (**C-D**) The representative images of the clones expressing the indicated transgenes (GFP^+). Scale bar, 20 µm. (**E**) The percentage of GFP^+ area in the disc area. n=9 (*Ns1 RNAi+ w RNAi*), 8 (*Ns1 RNAi+ Rheb RNAi*). (**F**) The fraction of $cDcp1^+$ cells within the GFP^+ clones. n=71 clones from 15 discs (*Ns1 RNAi+ w RNAi*), 77 clones from 16 discs (*Ns1 RNAi+ Rheb RNAi*). The genotypes of samples used in this figure are listed in Table S4.

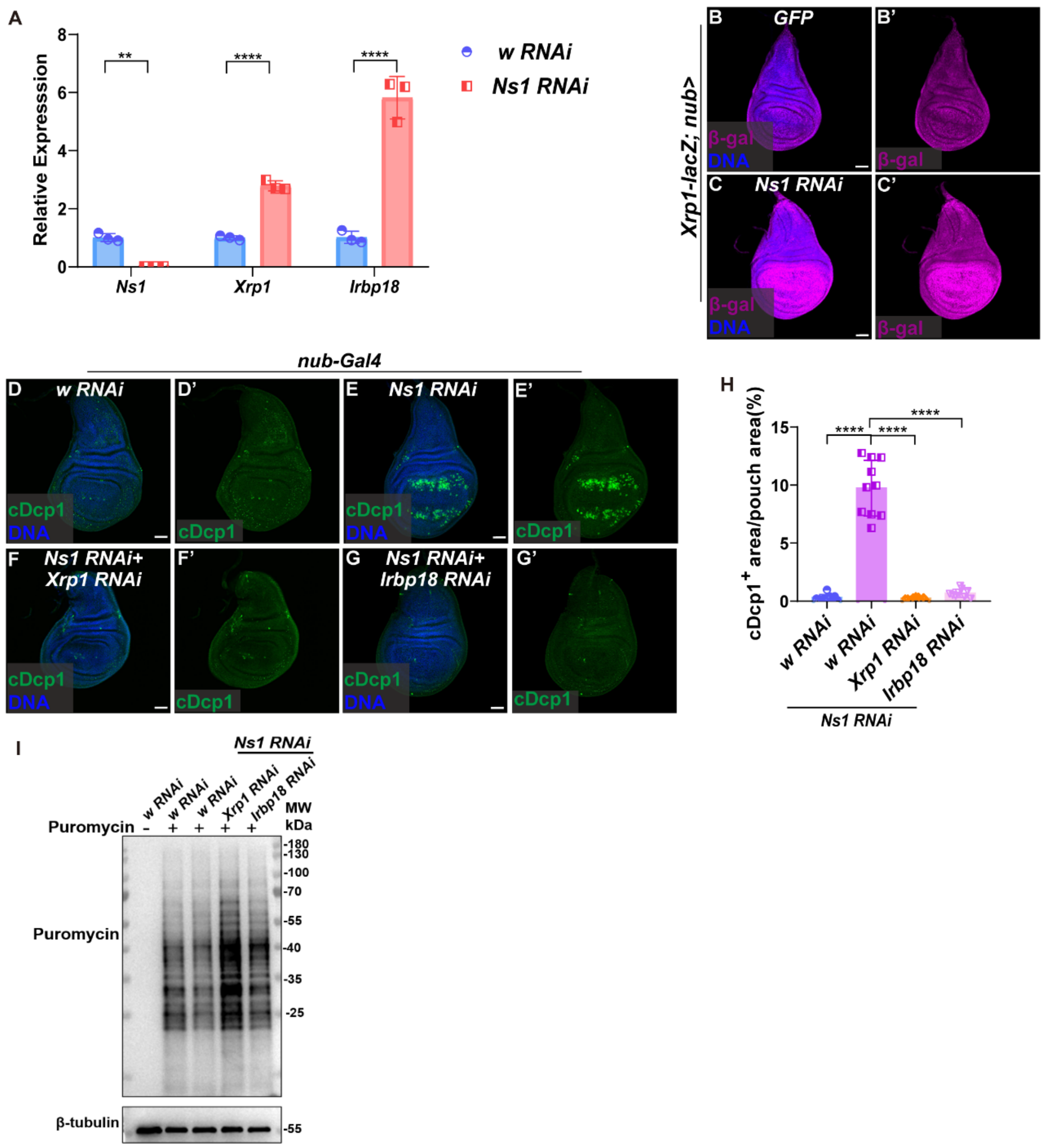

Fig S6. Ns1 deficiency induces apoptosis by upregulating the Xrp1/Irbp18 complex. (**A**) The relative mRNA levels of *Ns1*, *Xrp1* and *Irbp18* in wing discs with *w RNAi* or *Ns1 RNAi* expressed in the whole wing discs using *act-Gal4*. The average mRNA level in *act> w RNAi* group was set as 1. n= 3. Each biological replicate was prepared from ~100 wing discs. (**B-C**) The representative images of *Xrp1-lacZ* expression in wing discs with the indicated transgenes expressed in the pouch under the control of *nub-Gal4*. Scale bar, 20 µm. (**D-G**) The representative images of cDcp1 staining in wing discs with the indicated transgenes expressed in the pouch under the control of *nub-*

Gal4. Scale bar, 20 µm. (**H**) The fraction of cDcp1^{+} area in the pouch area. n= 9 (*w RNAi*), 10 (*Ns1 RNAi+ w RNAi*), 9 (*Ns1 RNAi+ Xrp1 RNAi*), 9 (*Ns1 RNAi+ Irbp18 RNAi*). (**I**) Western blots showing puromycin incorporation in the indicated discs. Each sample was prepared from ~100 discs. **: *P*< 0.01; ****: *P*< 0.0001. The genotypes of samples used in this figure are listed in Table S4.

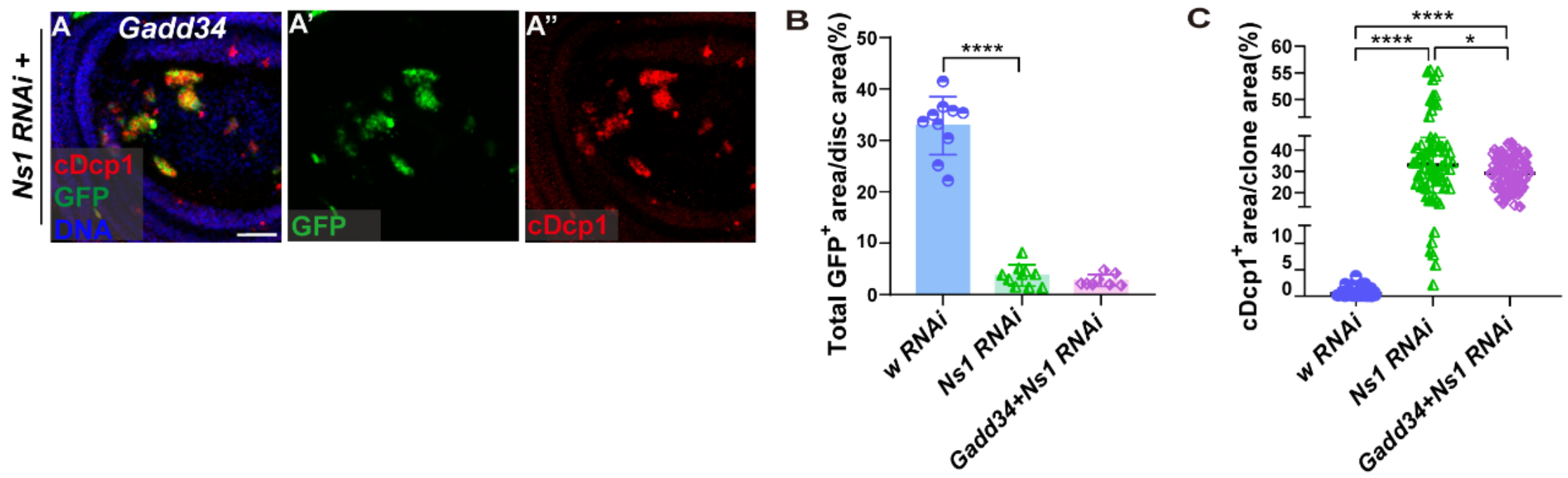

Fig S7. Inhibiting eIF2α phosphorylation does not suppress apoptosis in Ns1-deficient clones.

(**A**) The representative images of cDcp1 staining in discs with *Ns1 RNAi* and *Gadd34* co-expressed in the GFP^+ clones. Scale bar, 20 μm. (**B**) The percentage of GFP^+ area in the disc area. n=10 (*w RNAi*), 10 (*Ns1 RNAi*), 8 (*Ns1 RNAi*+ *Gadd34*). (**C**) The fraction of $cDcp1^+$ cells within the GFP^+ clones. n=79 clones from 13 discs (*w RNAi*), 71 clones from 15 discs (*Ns1 RNAi*), 80 clones from 13 discs (*Ns1 RNAi*+ *Gadd34*). *: $P< 0.05$; ****: $P< 0.0001$. The genotypes of samples used in this figure are listed in Table S4.

Table S1. RPs identified in proteomics

Gene Name	*Ns1 RNAi* /*w RNAi* Ratio	*P* value	Change
**RpS**			
RpS30	0.371004	3.08E-06	Down
RpS23	0.375079	4.5E-06	Down
RpS27	0.390036	1.14E-05	Down
RpS17	0.403235	3.31E-05	Down
RpS25	0.41048	2.25E-06	Down
RpS18	0.418687	2.72E-07	Down
RpS6	0.436745	1.18E-06	Down
RpS9	0.438705	2.27E-06	Down
RpS13	0.453449	0.000124	Down
RpS20	0.479237	0.00014	Down
RpS3A	0.484003	6.04E-06	Down
RpS11	0.485556	1.17E-05	Down
RpS4	0.493376	1.22E-05	Down
RpS10b	0.493412	3.66E-05	Down
RpS14a	0.498366	2.46E-06	Down
RpS19a	0.503595	7.48E-06	Down
RpS3	0.514446	6.09E-06	Down
RpS16	0.516374	1.04E-05	Down
RpS8	0.53157	2.39E-05	Down
RpS21	0.549502	0.001634	Down
RpS24	0.554657	0.002505	Down
RpS29	0.566203	1.15E-05	Down
RpS2	0.58702	0.00018	Down
RpS7	0.623583	0.000128	Down
RpS28b	0.666683	0.000937	Down
RpS12	0.674444	7.31E-05	None
RpS15Aa	0.67798	0.005299	None
RpS15	0.71545	0.000381	None
RpS5a	0.783128	0.00144	None
RpS27A	0.907232	0.139848	None
RpS19b	0.90859	0.010135	None
RpS5b	0.919719	0.072701	None
**RpL**			
RpL34a	0.001	0.001	Down

RpL12	0.124724	0.064548	Down
RpL29	0.319029	1.89E-05	Down
RpL35	0.385201	0.000104	Down
RpL9	0.393371	1.18E-07	Down
RpL24	0.395934	3.62E-07	Down
RpL30	0.396551	1.51E-06	Down
RpL13A	0.438902	9.88E-05	Down
RpL31	0.444935	1.07E-05	Down
RpL13	0.452917	3.91E-07	Down
RpL37A	0.463389	4.96E-06	Down
RpL35A	0.469775	2.35E-05	Down
RpL19	0.470624	7.15E-06	Down
RpL22	0.473828	2.45E-08	Down
RpL14	0.474047	1.62E-05	Down
RpL28	0.481196	2.86E-05	Down
RpL38	0.488762	1.61E-05	Down
RpL4	0.492575	4.78E-05	Down
RpL21	0.494017	2.19E-06	Down
RpL36	0.496064	2.81E-06	Down
RpL37-1	0.498772	5.58E-05	Down
RpL23A	0.502303	4.8E-07	Down
RpL7	0.504201	9.13E-07	Down
RpL6	0.505285	2.16E-06	Down
RpL23	0.507432	0.004701	Down
RpL15	0.523237	3.97E-06	Down
RpL7A	0.524826	1.4E-05	Down
RpL27	0.532923	3.3E-06	Down
RpL5	0.536276	0.000114	Down
RpL10	0.538437	4.34E-06	Down
RpL26	0.545402	0.00016	Down
RpL27A	0.566452	0.000546	Down
RpL10Ab	0.567721	7.56E-05	Down
RpL36A	0.576789	1.94E-05	Down
RpL18A	0.580852	2.67E-05	Down
RpL3	0.583168	1.55E-05	Down
RpL18	0.596264	4.68E-05	Down
RpL11	0.641851	0.000974	Down

RpL8	0.659587	0.00073	Down
RpL32	0.665383	0.002326	Down
RpLP0	0.690328	0.000115	None
RpL17	0.753718	0.680819	None
RpLP1	0.762172	0.015018	None
RpLP2	1.196818	0.001172	None
RpLP0-like	1.599594817	6.1E-05	Up
RpL7-like	1.786913	0.00032	Up
RpL24-like	2.331589	5.05E-08	Up

Table S2. The oligonucleotides used in this study.

Primer	Forward (5'-3')	Reverse (5'-3')
**Construct *pJFRC-20×UAS-Ns1: GFP***		
Ns1: GFP	GCGGCCGCGGCTCGAGATGGCTTTAAAAAGGTTGAAG	CTTTACTCATGGATCCTTCAATCACATAGTCCTCATC
**qPCR**		
Actin5C	CAGATCATGTTCGAGACCTTCAAC	ACGACCGGAGGCGTACAG
ITS1	GAAGAAACAAAATTCGAAAG	CGTATGCCCATAACTAAGAT
ITS2	AGACTATGCTAATTAAGTTGC	ATGCTAGACATTTCTCATAT
18S	CTCATATCCGAGGCCCTGTA	ACGAACGTTTTAACCGCAAC
28S	CGCTACGTCCGTTGGATTAT	CAATGCAAATTGCCCCTTAT
Ns1	CTTTAAGGCGGAACGCGAAC	TTGGTCACGGCGTTCTTGTA
Xrp1	ACATACTCGTCGCCCAACTG	ACATACTCGTCGCCCAACTG
Irbp18	AATCGGCCGAGGAACGTAAG	GATGATTCGATGGCCGTCCT
RpS4	CGAGGTCACCAAGATCGTCAT	GACCAGACGGAAGAACTCACC
RpS6	TGAAGCAGGGTGTCTTGACC	ACGCACAGACTTGCGCTTA
RpS9	AGCGTCTGTTCCAGGGTAATG	CACGTAATCGAGCTTCATGCG
RpS13	ATGGGTCGTATGCACGCTC	CAGCCAGGATGGGACAGTG
RpS18	TGACCGCCATCAAGGGAGT	CGCTTGGTAAGATCGACATCG
RpL3	GTTGGCACACCAAGAAGCTG	ATCTCGGTACGGTGGTGGTA
RpL4	CAGCTCTGGTCCAGTCCAAG	GACGCAGGAAGATGACAGCC
RpL9	CAGTGCGTGAAGATCCCTAAG	CCAACTCCTTCTTGGTTCCGA
RpL13	GTGGTCGAGTTCCGTGAGG	CCTTCTTGGGGTCTCCCTT

RpL19	TGAAATCAACGAGATCGCTAACA	CTTTCCGAATCCGCAGTGAC
RpL21	TTTCCCGTCCCTTCCGAAAG	TGTCCCTTAATGTCAACGATGT
RpL23	ACCCAAAAAGCCAACCGAGA	GCCGGAGCAGCCTTCTTAG
RpL24	GCATTCAGCGGGTACAAAATCT	GTCCAGGAAGGTGAACGACTT
RpL30	ATGGTGGCCGTTAAGAAACAA	GCAGTATTTGCCGGACTTCAT

Table S3. The antibodies used in this study.

Antibody Name	Company	Cat. No	Application	Dilution
cDcp1	Cell Signaling Technology	9578	IF	1:200
RpS6	ABclonal	A11874	IF, WB	1:400
Flag	Vazyme	RA1003	IF	1:400
β-Galactosidase	Promega	Z3783	IF	1:100
Ubiquitin	Santa Cruz Biotechnology	sc8017	IF	1:100
p-eIF2α	Cell Signaling Technology	3398	IF	1:200
RpS13	ABclonal	A15720	WB, IF	1:1000
RpL3	ABclonal	A6973	WB	1:1000
RpL4	Santa Cruz Biotechnology	sc-100838	WB	1:1000
p-RpS6	Santa Cruz Biotechnology	sc-293144	WB	1:1000
S6K	Selleck	F3527	WB	1:1000
p-S6K	Cell Signaling Technology	9209	WB	1:1000
GAPDH	Proteintech	60004-1-Ig	WB	1:5000
β-tubulin	Abways	AB2002	WB	1:10000
puromycin	ABclonal	A23031	WB	1:2000
568-goat anti-Rabbit	Thermo Fisher Scientific	A11011	IF	1:200
568-goat anti-mouse	Thermo Fisher Scientific	A11031	IF	1:200
488-goat anti-Rabbit	Thermo Fisher Scientific	A11008	IF	1:200
488-goat anti-mouse	Thermo Fisher Scientific	A11029	IF	1:200
647-goat anti-mouse	Thermo Fisher Scientific	A21235	IF	1:200
HRP-goat anti-rabbit	ORIGENE	ZB-2301	WB	1:5000
HRP-goat anti-mouse	ORIGENE	ZB2305	WB	1:5000

Table S4. Genotypes of the samples used in each figure.

Figure number/panel	Genotype
1A	*nub-Gal4/+; UAS-w RNAi/+*
1B, S6E	*nub-Gal4/UAS-Ns1RNAi (II)*
1C	*nub-Gal4/+; UAS-Ns1RNAi (III)/+*
1E, 2D, 3D, 6A, S3A	*hs-FLP/+; ex-lacZ/+; act>CD2>Gal4 UAS-GFP/UAS-w RNAi*
1F, 2E, 3E, S3B	*hs-FLP/+; ex-lacZ/UAS-Ns1 RNAi; act>CD2>Gal4 UAS-GFP/+*
1G, 2F, 3F, S3C	*hs-FLP/+; ex-lacZ/+; act>CD2>Gal4 UAS-GFP/UAS-Ns1 RNAi*
1L, S2A, 5H	*hs-FLP/+; UAS-GFP/UAS-Ns1 RNAi; act>CD2>Gal4 UAS-GFP/+*
1M, 2G, S3D	*hs-FLP/+; UAS-Ns1: GFP/+; act>CD2>Gal4 UAS-GFP/ UAS-Ns1 RNAi*
1N, 2I, 3G, S3E	*hs-FLP/+; UAS- miRGH/+; act>CD2>Gal4 UAS-GFP/UAS-Ns1 RNAi*
2A, 2C, 2J, 3C, S4, S6A	*act-Gal4/+; UAS-w RNAi/tubGal80ts* and *act-Gal4/+; UAS-Ns1 RNAi/tubGal80ts*
2H, 6C, S3F	*hs-FLP/+; UAS- p35/UAS-Ns1 RNAi; act>CD2>Gal4 UAS-GFP/+*
2K	*nub-Gal4/UAS-p35; UAS-w RNAi/UAS-Ns1 RNAi*
2L	*nub-Gal4/UAS-p35; UAS-Rpn1 RNAi/UAS-Ns1 RNAi*
3I	*nub-Gal4/+; UAS-w RNAi/+ and nub-Gal4/+; UAS-Ns1 RNAi/+*
4A	*hs-FLP/+; If/UAS-GFP; act>CD2>Gal4 UAS-mCD8: GFP/Xrp1-lacZ*
4B	*hs-FLP/+; If/UAS-Ns1 RNAi; act>CD2>Gal4 UAS-mCD8: GFP/Xrp1-lacZ*
4C, 6B, S5C	*hs-FLP/+; ex-lacZ/UAS-Ns1 RNAi; act>CD2>Gal4 UAS-GFP/UAS-w RNAi*
4D, 4H, 4L, 6E	*hs-FLP/+; ex-lacZ/UAS-Ns1 RNAi; act>CD2>Gal4 UAS-GFP/UAS-Xrp1 RNAi*
4E, 4I, 4M, 6F	*hs-FLP/+; ex-lacZ/UAS-Ns1 RNAi; act>CD2>Gal4 UAS-GFP/UAS-Irbp18 RNAi*
4J	*hs-FLP/+; RpL3: 3×Flag/act>CD2>Gal4 UAS-GFP; UAS-Ns1 RNAi/UAS-Xrp1 RNAi*
4K	*hs-FLP/+; RpL3: 3×Flag/act>CD2>Gal4 UAS-GFP; UAS-Ns1 RNAi/UAS-Irbp18 RNAi*
5A	*hs-FLP/+; TRE: DsRed/act>CD2>Gal4 UAS-GFP; MKRS/UAS-w RNAi*

5B	*hs-FLP/+; TRE: DsRed/UAS-GFP; act>CD2>Gal4 UAS-GFP/UAS-Ns1 RNAi*
5C	*hs-FLP/+; TRE: DsRed/UAS-Ns1: GFP; act>CD2>Gal4 UAS-GFP/UAS-Ns1 RNAi*
5D	*hs-FLP/+; TRE: DsRed/act>CD2>Gal4 UAS-GFP; UAS-puc/UAS-Ns1 RNAi*
5E	*hs-FLP/+; TRE: DsRed/act>CD2>Gal4 UAS-GFP; UAS-Ns1 RNAi/UAS-Xrp1 RNAi*
5F	*hs-FLP/+; TRE: DsRed/act>CD2>Gal4 UAS-GFP; UAS-Ns1 RNAi/UAS-Irbp18 RNAi*
5G	*hs-FLP/+; UAS-Ns1 RNAi/act>CD2>Gal4 UAS-GFP; UAS-puc/Xrp1-lacZ*
5I, 5L	*hs-FLP/+; act>CD2>Gal4 UAS-GFP/UAS-Ns1 RNAi; UAS-puc/+*
6D, 6H, S7	*hs-FLP/+; ex-lacZ/UAS-Gadd34; act>CD2>Gal4 UAS-GFP/UAS-Ns1 RNAi*
S1A	*MS1096-gal4/+; UAS-w RNAi/+*
S1B	*MS1096-gal4/+; UAS-Ns1RNAi (II)/+*
S1C	*MS1096-gal4/+; UAS-Ns1RNAi (III)/+*
S1D	*MS1096/+; UAS-Ns1: GFP/+; UAS-Ns1 RNAi/+*
S2B	*hs-FLP/+; UAS-Ns1 RNAi/act>CD2>Gal4 UAS-GFP; UAS-p53.DN/+*
S3G	*hs-FLP/+; ex-lacZ/RpL3: 3×Flag; act>CD2>Gal4 UAS-GFP/UAS-w RNAi*
S3H	*hs-FLP/+; ex-lacZ/RpL3: 3×Flag; act>CD2>Gal4 UAS-GFP/UAS-Ns1 RNAi*
S3I	*hs-FLP/+; RpL3:3×Flag/UAS-Ns1: GFP; act>CD2>Gal4 UAS-GFP/UAS-Ns1 RNAi*
S3J	*hs-FLP/+; RpL3:3×Flag/UAS-p35; act>CD2>Gal4 UAS-GFP/UAS-Ns1 RNAi*
S5A, S5B	*act-Gal4/+; UAS-w RNAi/tubGal80ts* and *act-Gal4/+; UAS-Rheb RNAi/tubGal80ts*
S5D	*hs-FLP/+; ex-lacZ/UAS-Ns1 RNAi; act>CD2>Gal4 UAS-GFP/UAS-Rheb RNAi*
S6B	*nub-Gal4/UAS-GFP; Xrp1-lacZ/+*
S6C	*nub-Gal4/UAS-Ns1 RNAi; Xrp1-lacZ/+*
S6D	*nub-Gal4/+; UAS-w RNAi/+*
S6F	*nub-Gal4/UAS-Ns1 RNAi; UAS-Xrp1 RNAi/+*
S6G	*nub-Gal4/UAS-Ns1 RNAi; UAS-Irbp18 RNAi/+*

S6I	*nub-Gal4/+; UAS-w RNAi, nub-Gal4/UAS-Ns1 RNAi; UAS-w RNAi/+, nub-Gal4/UAS-Ns1 RNAi; UAS-Xrp1 RNAi/+, nub-Gal4/ UAS-Ns1 RNAi;UAS-Irbp18 RNAi/+*

Dataset S1. Proteomic data.

The differentially expressed proteins, GO terms enriched in genes downregulated or upregulated in *Ns1* knockdown discs, KEGG terms enriched in genes downregulated or upregulated in *Ns1* knockdown discs were listed in different tabs.
