# Supplementary material for: Loss of Drosophila nucleostemin 1 disrupts ribosomal protein homeostasis and rRNA processing to trigger apoptosis via the Xrp1/Irbp18 complex
Source: Cell Death Discov. 2026 Jun 14;12:342. doi: 10.1038/s41420-026-03205-9 (PMC13490407; doi:10.1038/s41420-026-03205-9)

Fig2C

RpL3
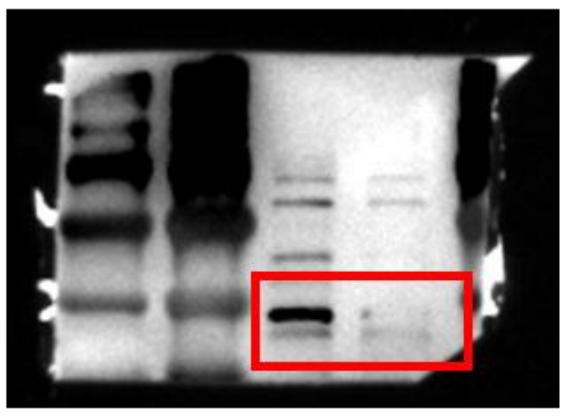

RpL4
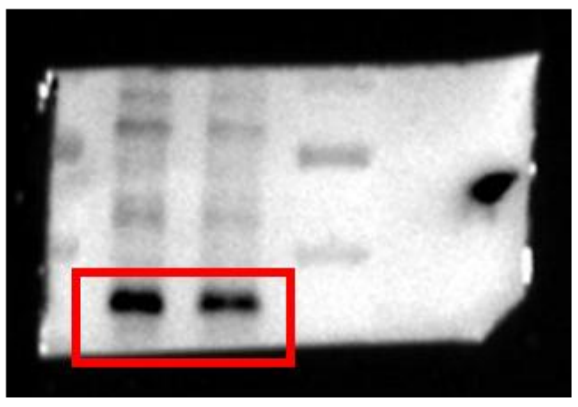

GAPDH
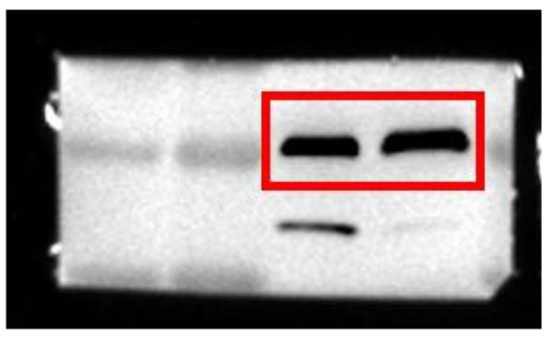

GAPDH
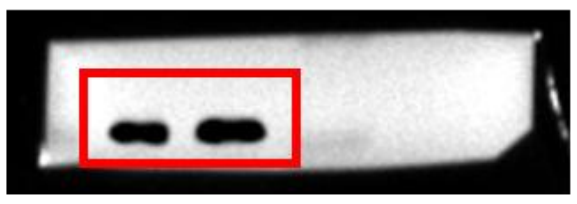

RpS13
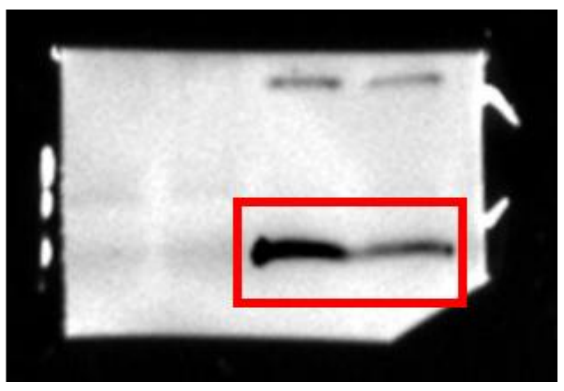

RpS6
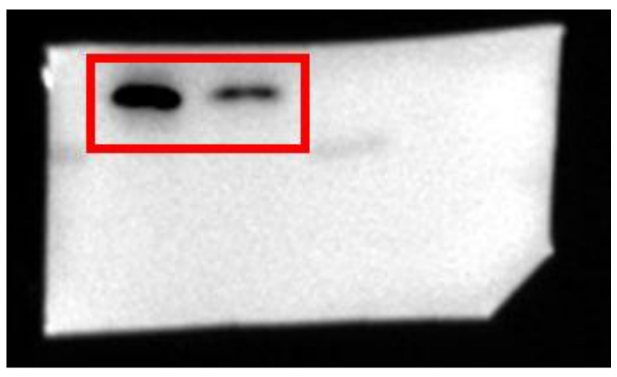

Fig3I

Puromycin
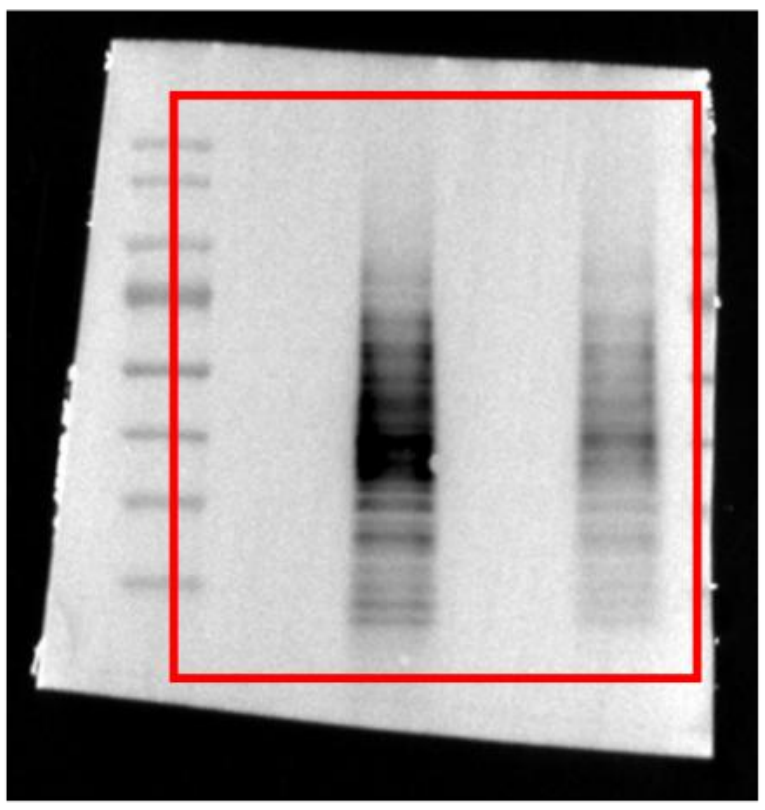

β-tubulin
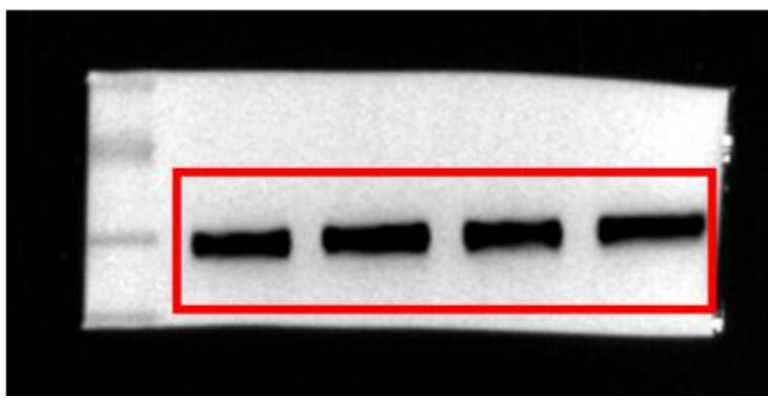

FigS5A

p-S6K

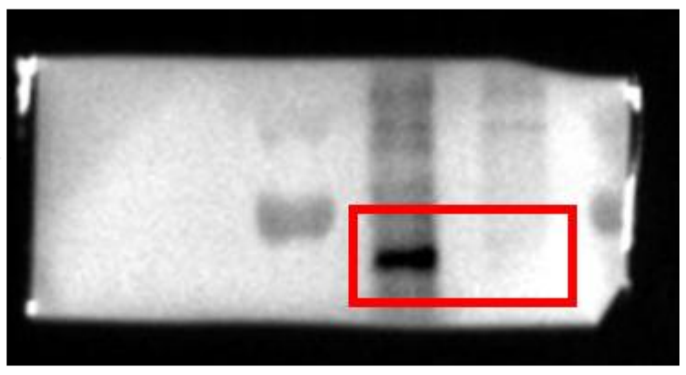

S6K

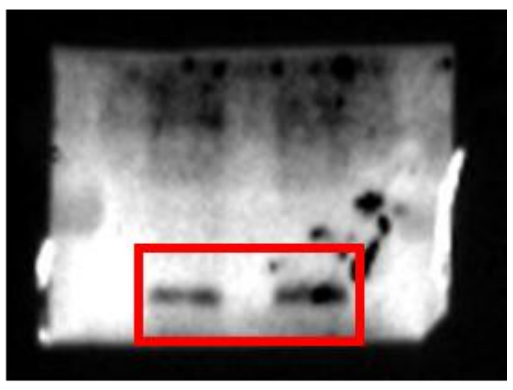

β-tubulin

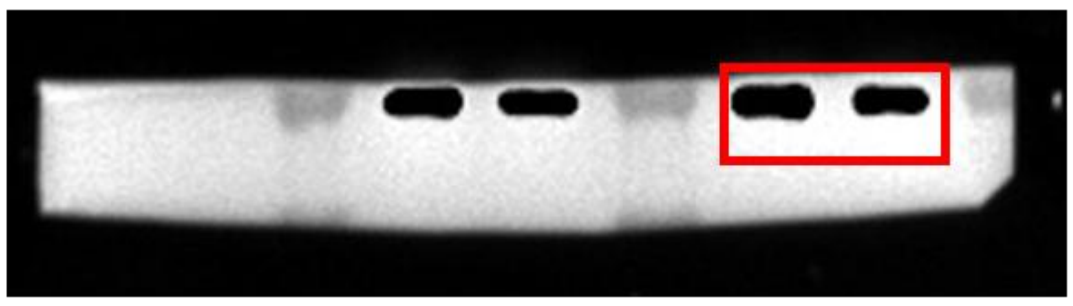

RpS6

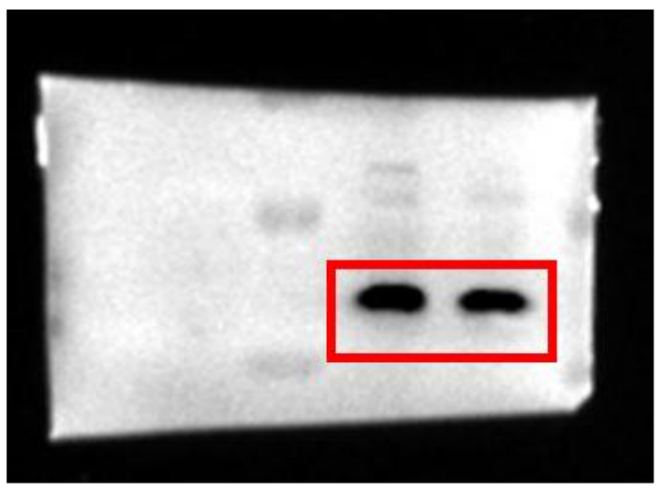

p-RpS6

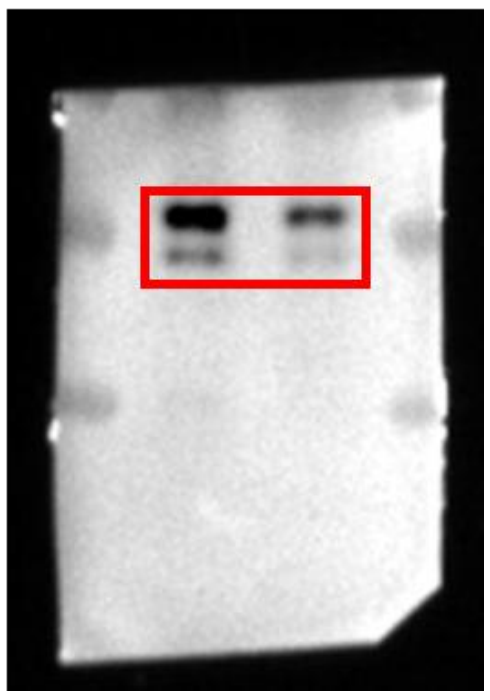

FigS5B

Puromycin

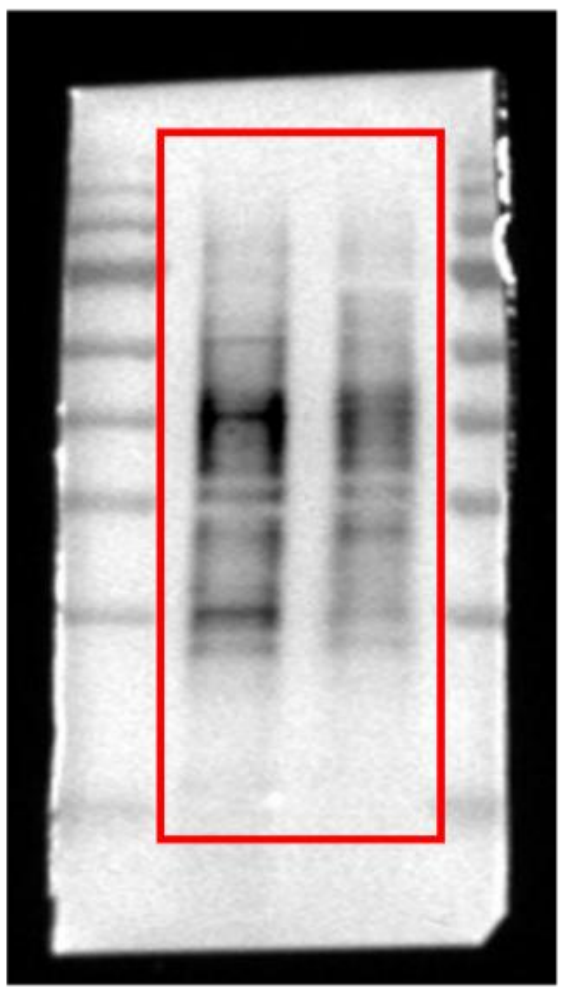

β-tubulin

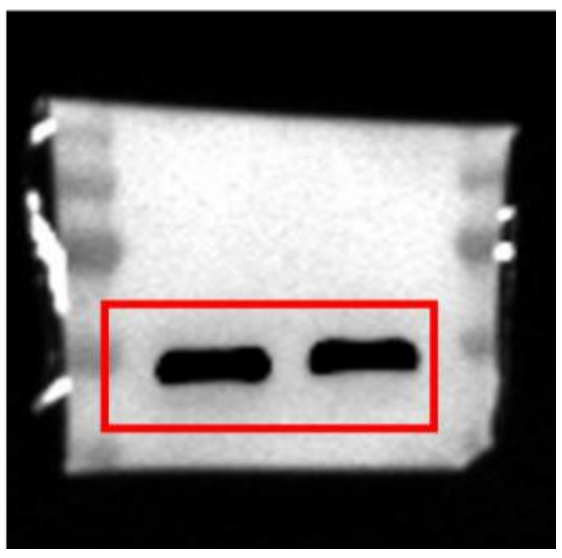

FigS6I

Puromycin

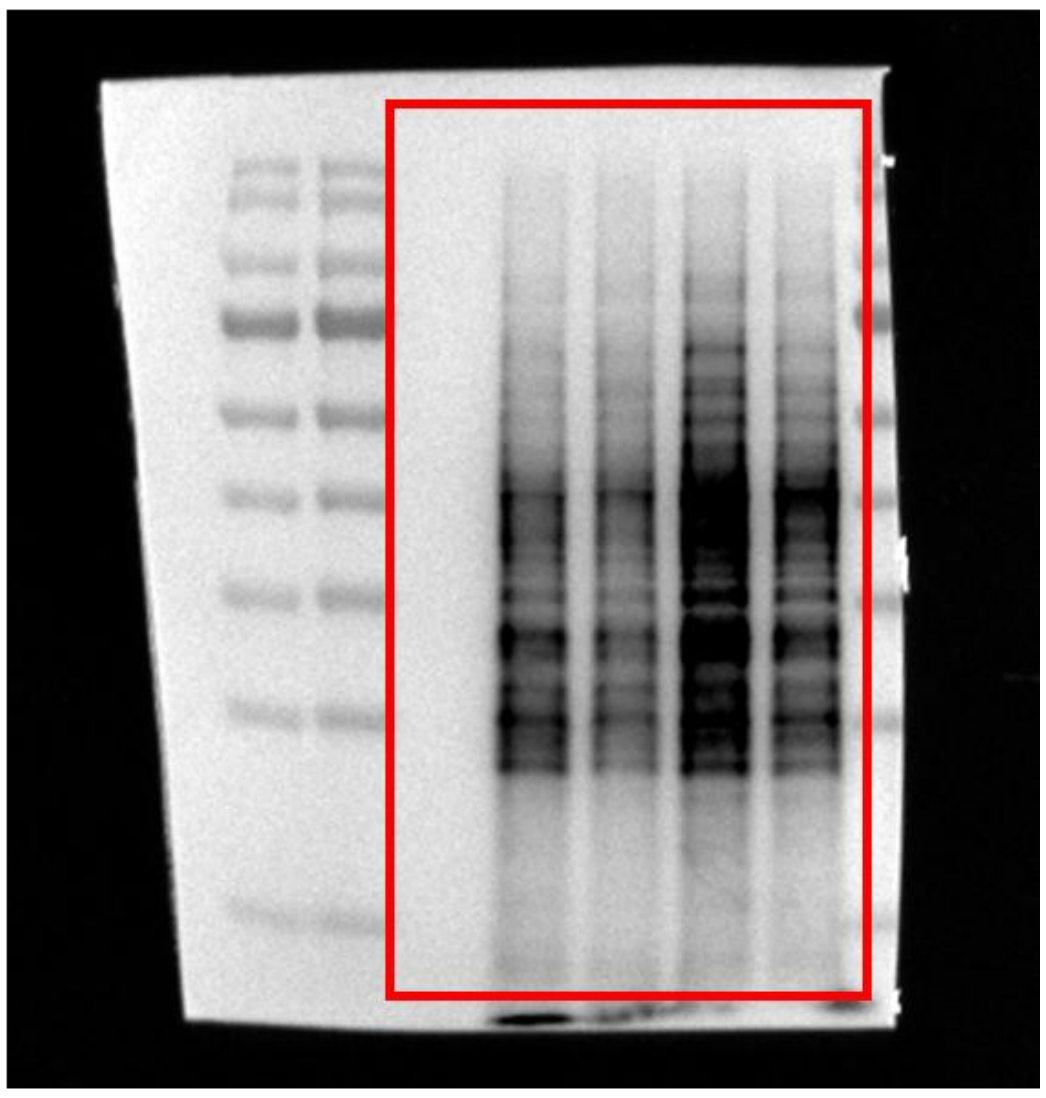

β-tubulin

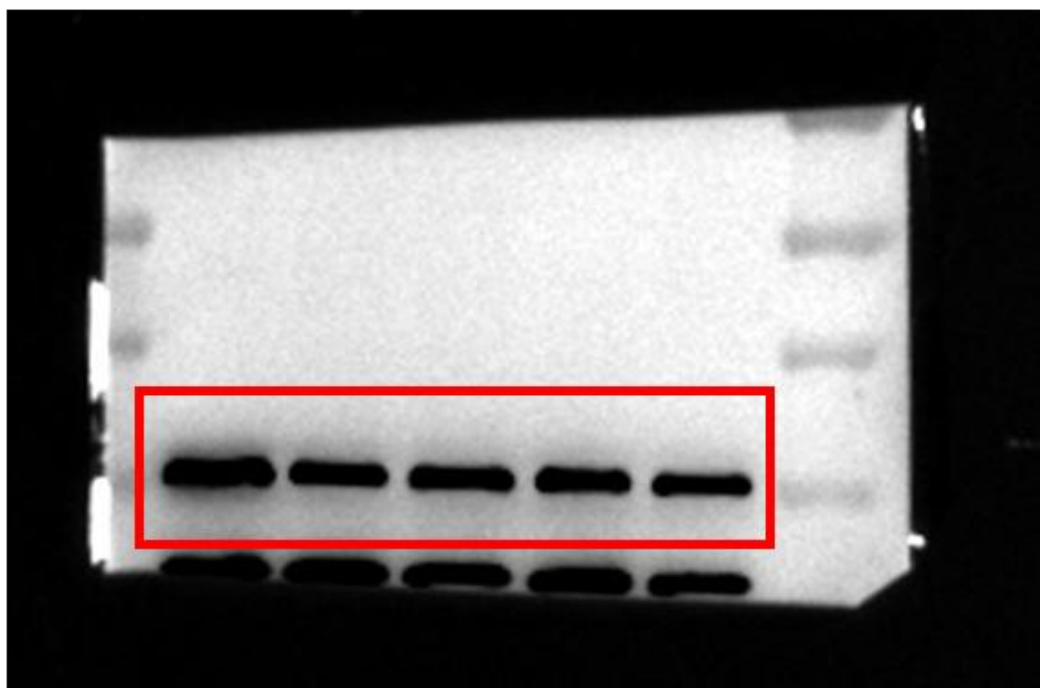

Supplement: Supplementary file 2 [file 41420_2026_3205_MOESM3_ESM.pdf]
